# Genome-wide landscape of runs of homozygosity and differentiation across Egyptian goat breeds

**DOI:** 10.1186/s12864-023-09679-6

**Published:** 2023-09-26

**Authors:** Ahmed M. Sallam, Henry Reyer, Klaus Wimmers, Francesca Bertolini, Adel Aboul-Naga, Camila U. Braz, Alaa Emara Rabee

**Affiliations:** 1 Animal and Poultry Breeding Department, Desert Research Center, Cairo, Egypt; 2 Research Institute for Farm Animal Biology (FBN), Wilhelm-Stahl-Allee 2, 18196 Dummerstorf, Germany; 3 Faculty of Agricultural and Environmental Sciences, University of Rostock, Justus-von-Liebig-Weg 6b, 18059 Rostock, Germany; 4 Department of Agricultural and Food Sciences, University of Bologna, Bologna, Italy; 5 Animal Production Research Institute, Agricultural Research Center, Dokki, Cairo Egypt; 6 Animal and Poultry Nutrition Department, Desert Research Center, Cairo, Egypt; 7 Department of Animal Sciences, University of Illinois Urbana-Champaign, 1207 Gregory Dr, Urbana, 61801 USA

**Keywords:** Adaptation, Local breeds, Selection signature, Inbreeding, Homozygosity

## Abstract

Understanding the genomic features of livestock is essential for successful breeding programs and conservation. This information is scarce for local goat breeds in Egypt. In the current study, genomic regions with selection signatures were identified as well as runs of homozygosity (ROH), genomic inbreeding coefficients (F_ROH_) and fixation index (F_ST_) were detected in Egyptian Nubian, Damascus, Barki and Boer goat breeds. A total of 46,268 SNP markers and 337 animals were available for the genomic analyses. On average, 145.44, 42.02, 87.90 and 126.95 ROHs were detected per individual in the autosomal genome of the respective breeds. The mean accumulative ROH lengths ranged from 46.5 Mb in Damascus to 360 Mb in Egyptian Nubian. The short ROH segments (< 2 Mb) were most frequent in all breeds, while the longest ROH segments (> 16 Mb) were exclusively found in the Egyptian Nubian. The highest average F_ROH_ was observed in Egyptian Nubian (~ 0.12) followed by Boer (~ 0.11), while the lowest F_ROH_ was found in Damascus (~ 0.05) and Barki breed (~ 0.03). The estimated mean F_ST_ was 0.14 (Egyptian Nubian and Boer), 0.077 (Egyptian Nubian and Barki), 0.075 (Egyptian Nubian and Damascus), 0.071 (Barki and Boer), 0.064 (Damascus and Boer), and 0.015 (Damascus and Barki), for each pair of breeds. Interestingly, multiple SNPs that accounted for high F_ST_ values were observed on chromosome 6 in regions harboring *ALPK1* and *KCNIP4*. Genomic regions overlapping both F_ST_ and ROH harbor genes related to immunity (*IL4R, PHF23, GABARAP, GPS2*, and *CD68*), reproduction (*SPATA2L, TNFSF12, TMEM95*, and *RNF17*), embryonic development (*TCF25* and *SOX15*) and adaptation (*MC1R, KDR*, and *KIT*), suggesting potential genetic adaptations to local environmental conditions. Our results contribute to the understanding of the genetic architecture of different goat breeds and may provide valuable information for effective preservation and breeding programs of local goat breeds in Egypt.

**Supplementary Information:**

The online version contains supplementary material available at 10.1186/s12864-023-09679-6.

## Introduction

Goat (*Capra hircus*) breeding is of high relevance in several countries because of their high adaptability to diverse environmental conditions compared to other livestock species [1]. Egypt has about 3.4 million live goats [1] that are mainly raised for meat production with minor importance for milk production (except in the coastal regions and oases) [2]. They constitute about 6% of the total red meat production in Egypt, in addition to socioeconomic impacts in rural areas as cash resources when needed [2]. Of all local breeds, Egyptian Nubian (also known as Zaraibi) has the highest potential for prolificacy and milk production [3] and is one of the progenitors of the Anglo-Nubian British breed [4]. Barki is a popular breed in the western desert of Egypt, while the other breeds are mainly present in the Nile Delta [5]. Damascus goat is considered one of the best dual-purpose breeds in the middle east, with high production performance [6] and it was introduced to Egypt more than 50 years ago to improve the productivity of local breeds by crossbreeding [2]. Besides, Boer goat is a transboundary breed that has been introduced to several countries worldwide, including Egypt, to improve the productivity of local breeds. Accordingly, it can by hypothesized that the genomes of Egyptian Nubian, Damascus and Boer goats have evolved for high production and reproductive performance under intensive breeding systems, while Barki has evolved to survive under harsh environmental conditions in desert regions [5].

In Egypt, Barki, Damascus, and Boer goat breeds are reared for meat production, while the Egyptian Nubian is usually reared for milk production [2]. Notably, the Egyptian Nubian has a wide range of coat colors, while Damascus and Barki have reddish brown and black coat colors, respectively. Boer goats are usually found in two colors, white body with reddish brown neck or completely reddish-brown. Pictures illustrating each breed included in the study are presented in Supplementary Figure S1.

Recently, the availability of genomic resources for this species as well as the development of high throughput genotyping tools allowed the exploration of the genomic architecture of local breeds for conservation and breeding purposes [7–9]. Concerns about local breeds arise from their relatively small population size and lack of proper breeding plans. Thus, mating of related individuals (i.e. inbreeding) increases, leading to a reduction in fitness due to increased homozygosity, which is referred to as inbreeding depression [7]. Therefore, several genome-wide approaches have been proposed to detect inbreeding in different goat breeds, such as the calculation of the genomic inbreeding coefficient (F_ROH_) [8–10].

Domestication and selective breeding are main factors for the high diversity of goat breeds, which left genomic footprints called “selection signatures” [11] with particular phenotypes of various economic importance [12]. Selection signatures can be defined as the reduction, elimination or change of genetic variation in genomic regions that are adjacent to causative variants in response to natural or artificial selection pressures [13]. Such variants usually affect several traits and contribute to the shaping of a breed [14–16]. Recently, two approaches have been widely used to detect signatures of selection in several goat breeds: Runs of homozygosity (ROH) and Fixation index (F_ST_) [5, 8–10]. Runs of homozygosity are long genomic stretches with a homozygous genotype that arise when two haplotypes share a recent common ancestor [16] and in an individual that has undergone the selection process [17, 18]. Runs of homozygosity provide useful population-level information on inbreeding characteristics and locations of selection signatures [19]. F_ST_ is a measurement of the differences in allele frequencies between populations [20]. The selection usually increases the frequency of a particular allele in one breed, thereby increasing the heterozygosity of certain loci in a population leading to higher genetic differentiation between breeds [21]. Notably, F_ST_ is more suitable for detecting selective events in the distant past, whereas ROH can detect recent selection signatures [22].

Detection of such important genome features in local breeds is important to understand their adaptation ability and to reduce inbreeding [23]. This has been widely applied using SNP arrays in cattle [18, 21, 22, 24, 25], buffaloes [26, 27], sheep [28] and goats [29, 30]. Detection approaches rely on screening the genomes for regions of homozygosity and estimating differences in allele or haplotype frequency between populations [23, 30]. So far, such information is still limited for local goat breeds in Egypt. Only one study conducted by Kim et al. [5] reported that the F_ROH_ and the average lengths of ROH were lower in Barki goats compared with exotic breeds, such as Boer. Moreover, higher proportion of individuals lacking long stretches of ROH was observed in Barki compared with the non-native breeds. Therefore, the objective of this study was to evaluate F_ROH_ and to detect signatures of selection through ROH analyses and F_ST_-based comparison of Egyptian Nubian, Damascus, Barki and Boer goat breeds, using SNP arrays. This will allow a better understanding of the genomic landscape and the evolutionary history of these breeds, which may be beneficial for future goat breeding programs.

## Materials and methods

### Study area and sample collection

All animal procedures included in the current study were approved by the Animal Breeding Ethics Committee at the Desert Research Center (DRC) in Egypt (Project ID: 43,213) with approval reference number AB/NO2020. Additionally, an informed consent was granted for Boer animals from the owners for the inclusion of Boer goats in the study.

**Fig. 1:**
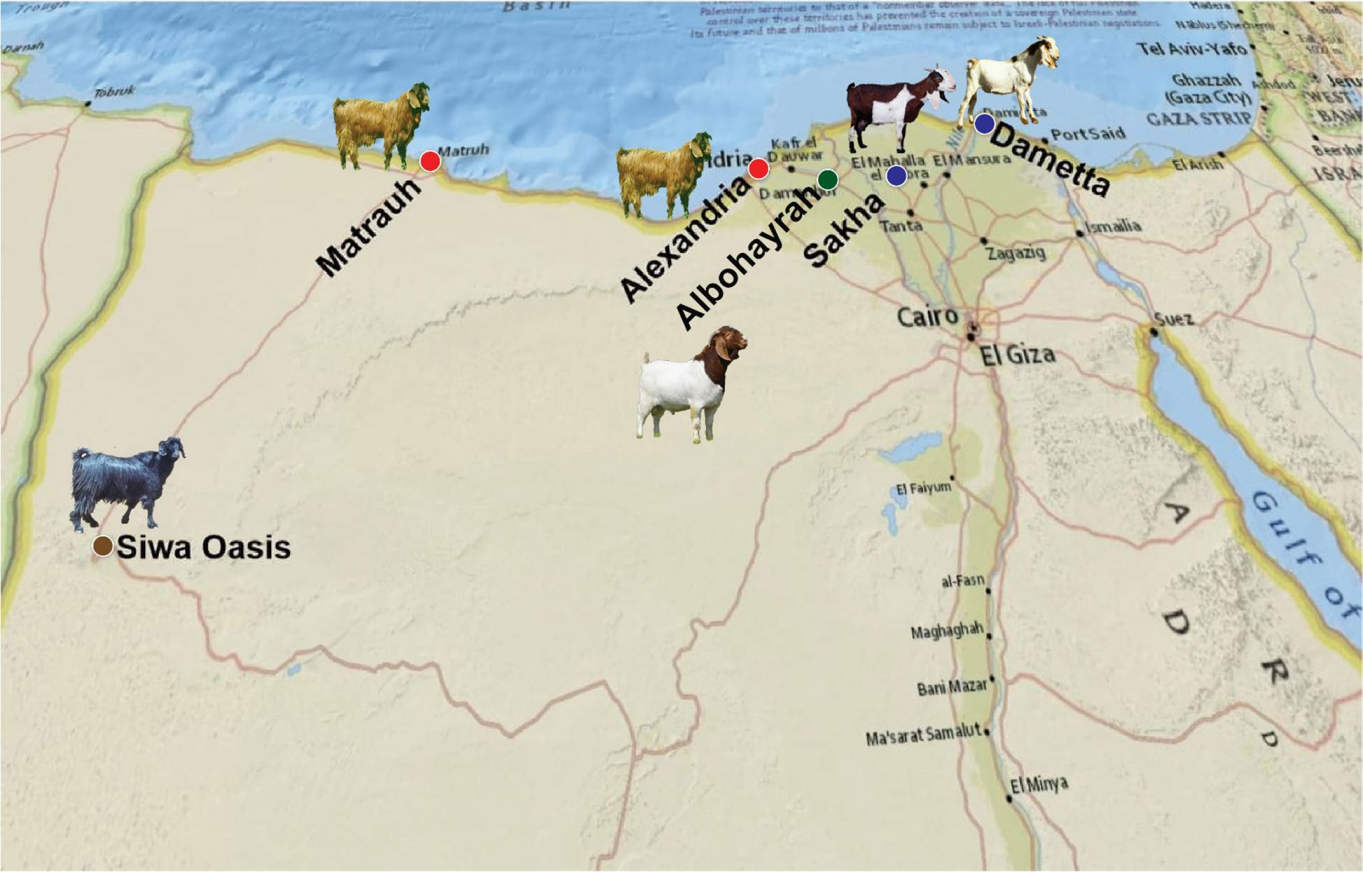
Map illustrating the area of sampling for Egyptian goat populations. The sampling locations are shown as circles coloured blue for Egyptian Nubian, red for Damascus, brown for Barki, and green for Boer

Four goat breeds were used in this study: Egyptian Nubian (*n* = 235), Damascus (*n* = 95), Barki (*n* = 28), and Boer (*n* = 26). The Egyptian Nubian, Damascus, and Barki animals belong to Serw, Maryout, and Siwa Research Farms, respectively, and the Boer animals were obtained from a commercial private farm in the North of Egypt (Fig. 1). Blood samples were collected from the jugular vein of each animal included in the study using vacutainer tubes containing EDTA. Samples were transferred in an icebox to the Molecular Genetics laboratory, Desert Research Center in Cairo for DNA extraction. Genomic DNA was extracted using a QIAamp DNA Blood Mini Kit provided by Qiagen® (Germany) following the manufacturer’s protocol.

### Genotyping, quality control and filtering

The quantity and quality of extracted DNA was assessed using the Nanodrop spectrophotometer. High-quality DNA samples (≥ 50 ng/µL) were genotyped at the Research Institute for Farm Animal Biology, Dummerstorf, Germany, using the Illumina®inc. Goat_IGGC_65K_v2 Infinium HD SNP chip (Illumina, San Diego, CA, USA). SNP locations reported in this paper are based on the genome version of *Capra hircus* available from the National Center for Biotechnology Information (ARS1.2, NCBI). The genotyping BeadChip contained 59,727 SNPs in total, evenly distributed throughout the caprine genome. Genotype calling was performed using GenomeStudio software (Illumina Inc., San Diego, CA, USA) according to the manufacturer’s protocols.

The whole dataset containing all genotyped individuals was filtered for quality control (QC) with PLINK v1.9 software [31], using the following parameters: (i) genotype call rate < 0.99 for markers and < 90% for individuals; (ii) SNPs located on unknown or the same chromosomal positions and SNPs on sex chromosomes were also excluded from the subsequent analyses. Deviation from Hardy–Weinberg Equilibrium (HWE) was not applied here as a filtration criterion because inbreeding is a possible reason for deviation, which may influence the analysis [32]. Linkage Disequilibrium (LD) pruning was not applied in this study because LD is related to various evolutionary forces (e.g. inbreeding, artificial and natural selection), which are investigated by the ROH analysis [33]. Likewise, the MAF filtering was not implemented in the current study to avoid ignoring large numbers of homozygous regions [34].

Principal component analysis (PCA) was performed using the common filtered SNPs using the ggplot2 package [35] implemented in RStudio software [36].

### Runs of homozygosity (ROH) detection

ROH were identified in each goat using the consecutive runs method of the R package *detectRUNS* [37] with the main following parameters: (1) the minimum number of consecutive SNPs in an ROH was 20; (2) the minimum ROH length was set to 250 kb; (3) the maximum gap between consecutive homozygous SNPs was 1 Mb; (4) the maximum number of opposite genotypes in the run was set to 1; and (5) the maximum number of missing genotypes allowed was 1. The sum of ROH values per goat was calculated and ROH estimates were classified into five categories for each goat: 0–2 Mb, 2–4 Mb, 4–8 Mb, 8–16 Mb and above 16 Mb [33]. For each breed, the numbers of ROH length categories and the proportion of ROH on each autosome were calculated. The consecutive method was preferred over the sliding window approach in order to avoid the detection of artificial ROH shorter than the window described above (20 SNPs) [38]. The total number of ROH, the average number of ROH per individual, the average ROH length, the number of ROH per breed per chromosome, and the number of ROH per length class of length were estimated for each breed in the study.

### Estimation of the genomic inbreeding coefficient (F_ROH_)

The genomic inbreeding coefficient (F_ROH_) is the estimation of the fraction of the genome in ROH, where identical-by-descent chromosome copies coalesce in a recent ancestor and ranges from 0 to 1 [39]. F_ROH_ was estimated as ROH-based inbreeding for each goat as follows [40]: *F*_*ROH*_*= L*_*ROH*_*/L*_*auto*_, where *L*_*ROH*_ is the total length of ROHs in the genome of each individual and *L*_*auto*_ is the total size of the autosomes of goat genome covered by SNPs, which was 2.46 Gb [41].

### Selection signatures

Selection signatures within each breed were investigated based on the estimation of the occurrences of ROH across the genome. The percentage of SNP occurrences (%) was calculated for each breed to identify the genomic regions with a high frequency of ROH (ROH hotspots) and were plotted against chromosomes in Manhattan plots using the qqman package [42] in R. In agreement with Jiang et al. [43], the threshold considered in the current study was 40% of ROH occurrence for each breed, including only the genomic regions that contained a minimum number of 20 consecutive SNPs.

Additionally, the pairwise comparisons between breeds were performed by calculating F_ST_ at each SNP. These values were calculated with the Weir and Cockerham F_ST_ [44] implemented in PLINK v1.9 [31].

### Functional annotation, candidate genes and gene enrichment analysis

For each ROH region above the threshold, functional annotation of genes was obtained from BioMart at the Ensembl Genome Browser (https://www.ensembl.org/biomart/martview) [45]. Gene function and protein domain were identified by the UniProt and OMIA (Online Mendelian Inheritance in Animals) and the GeneCards databases [46]. Likewise, the top 10 SNPs with the highest F_ST_ values in all pairwise comparisons were investigated for annotated genes in the goat reference genome (ARS1.2) release 102, within a region spanning ± 0.5 Mb from each SNP [47, 48]. We explored the genes that overlapped with the identified genomic intervals to determine the functional categories that are over-represented and, therefore, are likely under selection in the studied Egypt goat breeds. ShinyGO *v.* 0.77 [49] software was used to perform the functional enrichment analysis based on gene ontology (GO) [50] and the Kyoto Encyclopedia of Genes and Genomes (KEGG) pathways [51] against the goat gene set ontologies. The default parameters of the program were applied, and the results were adjusted to the false discovery rate (FDR < 0.05).

## Results

### Quality control filtering and population structure

**Fig. 2:**
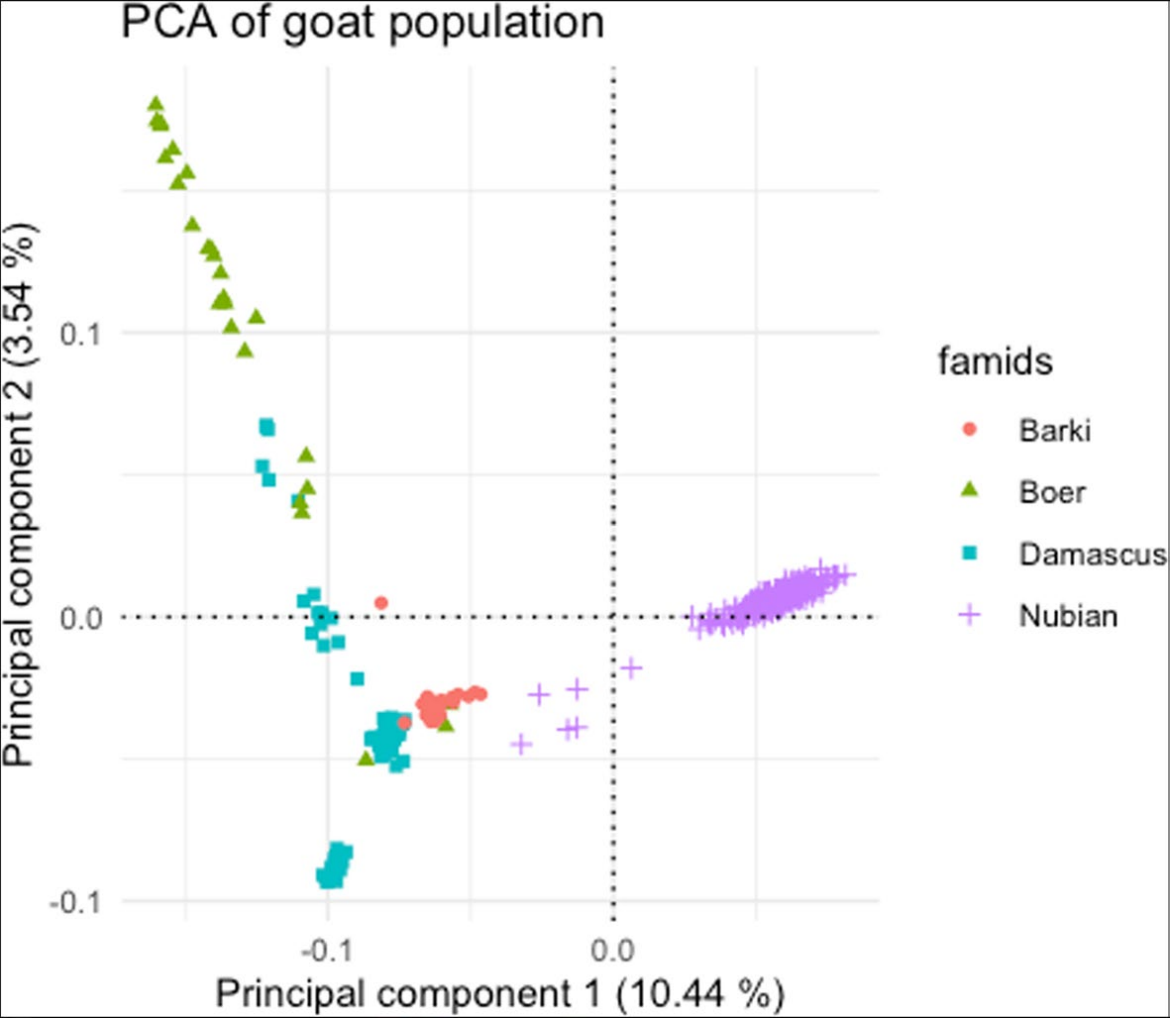
A principal component analysis (PCA) plot representing the genetic landscape of the studied goat population extended across the first and second components (PC1 and PC2) derived from eigenvectors and eigenvalues obtained from eigen decomposition of a genotypic (co)variance matrix between all individuals. The four breeds are presented in different colours, and each point represents one sample

The filtering steps removed 47 animals and 13,459 SNPs, leaving 46,268 variants and 337 animals (210 Egyptian Nubian, 82 Damascus, 21 Barki and 24 Boer). In the PCA, the first and the second principal components (Fig. 2) accounted for 10.44% and 3.54% of the genetic variation across all individuals of the four breeds, respectively. The PCA revealed that the studied breeds were clearly distinguished, with each breed representing a distinct cluster. A few individuals that may share common haplotypes across Damascus and Boer breeds and Damascus and Barki breeds were observed. The Egyptian Nubian individuals were isolated in a separate cluster.

### ROH statistics and F_ROH_ estimation

**Fig. 3:**
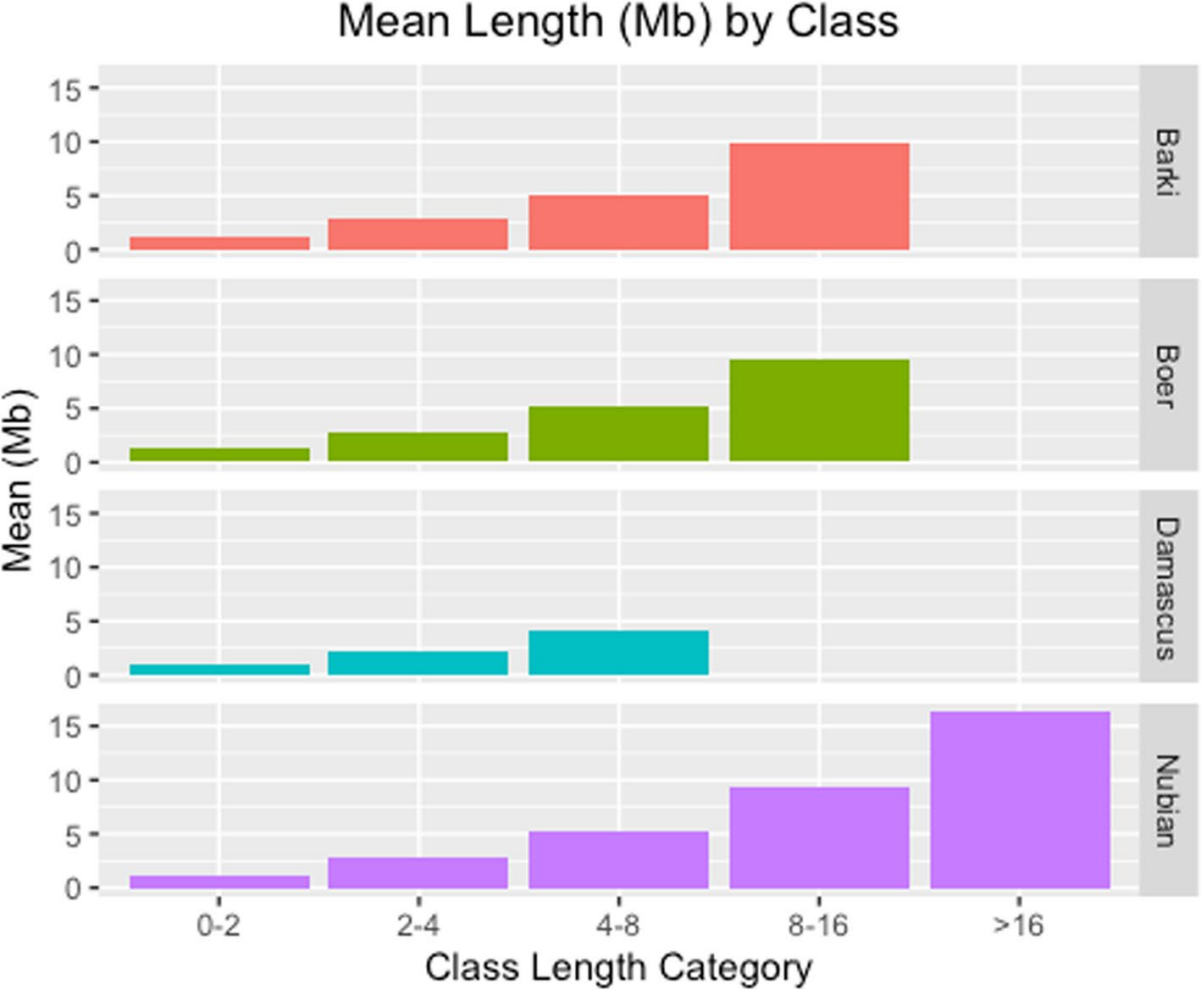
Runs of homozygosity distributed into 4 length classes in four Egyptian goat breeds (Egyptian Nubian, Damascus, Barki and Boer)

**Fig. 4:**
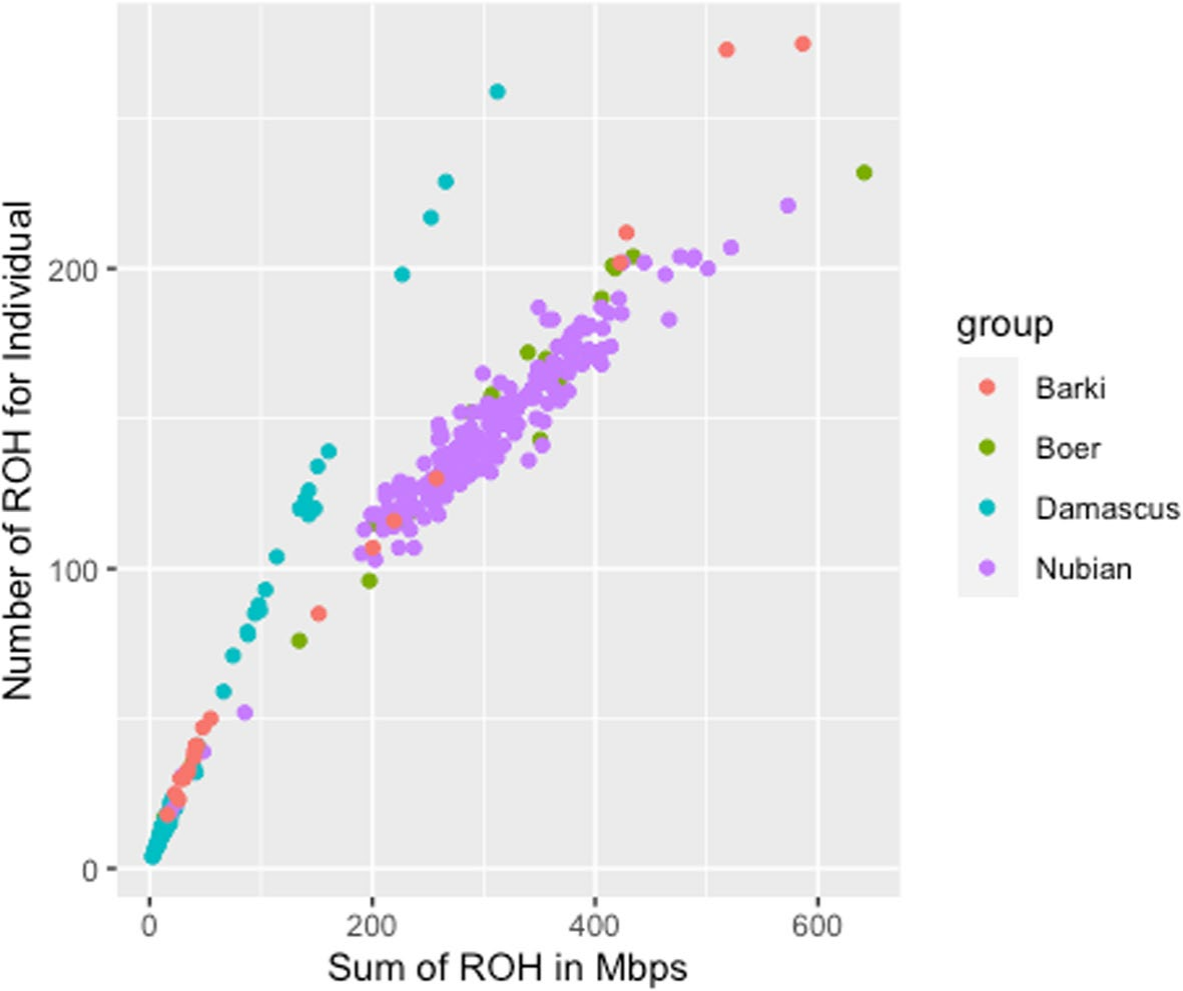
Patterns of runs of homozygosity (ROH) in the four Egyptian goat breeds (Egyptian Nubian, Damascus, Barki and Boer). Genomic coverage in ROH (x-axis) and ROH number per individual (y-axis)

The total number of ROH segments detected in Egyptian Nubian, Damascus, Barki and Boer were 30,544, 3446, 1846, and 3047 with an average number of 145.44, 42.02, 87.9, and 126.95, per individual, respectively. Mean ROH number and length in the studied goat population is presented in Table 1. Considering the length category distribution (Fig. 3), the shortest ROH segments (0–2 Mb) were the most common in all studied breeds. The percentage of ROH segments length less than 2 Mb were 71%, 64%, 96% and 63% in Egyptian Nubian, Damascus, Barki and Boer, respectively. The distribution of the 2–4 and 4–8 Mb ROH length classes were similar across the studied breeds. The frequencies of the long ROH segments (8–16 Mb) were found in Barki and Boer, while this class was absent in Damascus breed. The longest ROH segments (> 16 Mb) were only present in the Egyptian Nubian breed (0.01%). The ROH segments were identified in all breeds (Supplementary excel file), with mean accumulative lengths ranging from 46.5 Mb in Damascus to 306.0 Mb in Egyptian Nubian breed (Table 1). The maximum individual length of an ROH segment (16.33 Mb) and the maximum number of SNPs on a segment were found in Egyptian Nubian goats (*n* = 346 SNPs), while the Damascus breed displayed the lowest maximum values (4.1 Mb and 113 SNPs). Likewise, the mean accumulative ROH lengths were 154.4 and 261.7 in Barki and Boer, respectively. The individual patterns of ROH segment were similar in the Egyptian Nubian breed, while Barki and Boer showed a large individual variation in both ROH number and genomic coverage (Fig. 4).

**Table 1:** Mean ROH number and length in the studied goat population

Breed	Number	ROH number			ROH total length (Mb)		
		Mean	Min	Max	Mean^1^	Min	Max
Egyptian Nubian	210	145.44	19	251	306.0	18.9	573.1
Damascus	82	42.02	4	259	46.5	2.2	312.2
Barki	21	87.90	23	275	154.4	16.10	586.36
Boer	24	126.95	11	232	261.7	10.0	641.6

^1^Mean accumulative ROH length of all individuals in a breed in megabases; Min and Max are the minimum and maximum values for estimates observed in individual animals within the breeds, respectively. ROH = runs of homozygosity

**Fig. 5:**
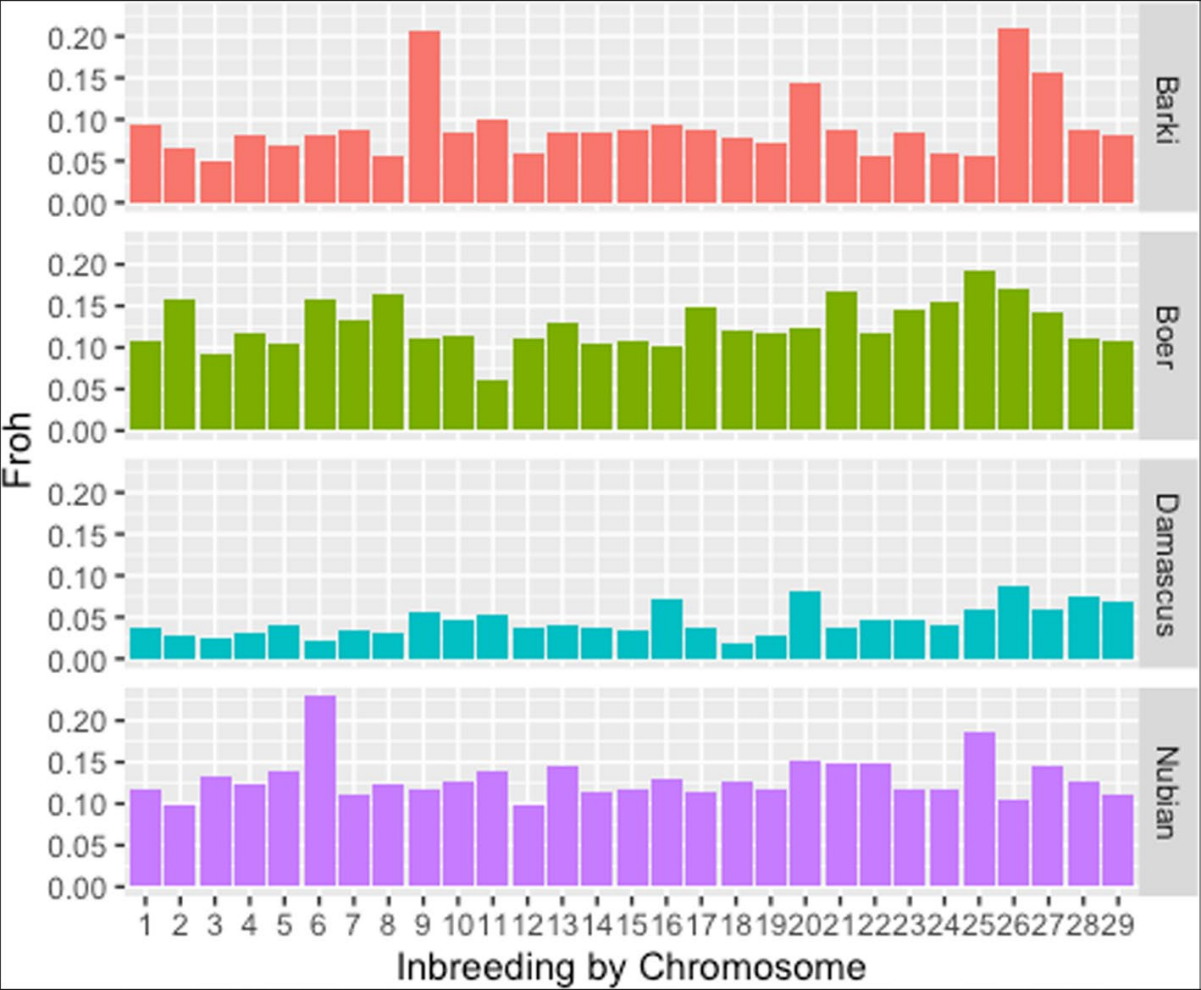
Inbreeding per chromosome estimated from the proportion of the chromosome covered by runs of homozygosity (ROH) for the 29 autosomes in four goat breeds in Egypt (Nubian, Damascus, Barki and Boer). F_ROH_ is the genomic inbreeding coefficient

The average values of F_ROH_ were 0.034, 0.057, 0.11 and 0.12 for Barki, Damascus, Boer and Egyptian Nubian breeds, respectively. The highest individual levels of F_ROH_ were found in Damascus (F_ROH_ = 0.36), Boer (F_ROH_ = 0.26), Barki (F_ROH_ = 0.18), and Egyptian Nubian breed (F_ROH_ = 0.23), while the lowest individual level of F_ROH_ were close to 0 and found in Boer goat (F_ROH_ = 0.0009). Inbreeding per chromosome estimated from the proportion of the chromosome covered by ROH for the 29 autosomes in the studied goat population is presented in Fig. 5. Across the studied goat population, chromosome 6 showed the highest F_ROH_ value (> 0.2) in Egyptian Nubian followed by chromosomes 9 and 26 in Barki (F_ROH_ ~ 0.2) and chromosome 25 in Boer (F_ROH_ <0.2), while chromosome 18 showed the lowest F_ROH_ value (< 0.05) in Damascus breed.

### ROH hotspot, functional annotation, candidate genes and gene enrichment analysis

**Fig. 6:**
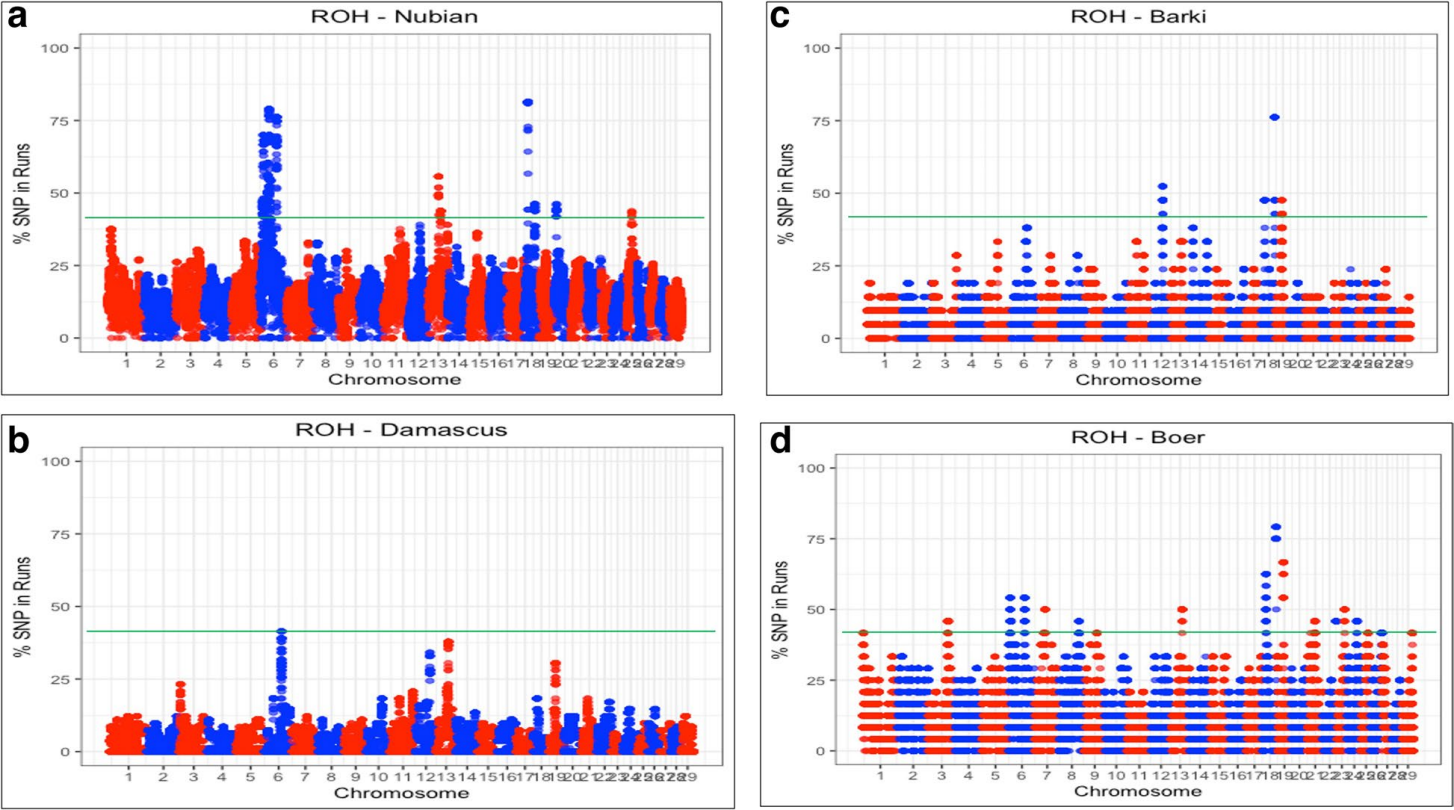
Manhattan plots of the distribution of ROH in the four goat breeds in Egypt (Egyptian Nubian, Damascus, Barki and Boer). The x-axis is the autosome number and the y-axis shows the frequency (%) at which each SNP was observed in ROH across individuals

The percentage of SNP occurrences was calculated for each breed to identify the genomic regions with a high frequency of ROH (ROH hotspots), potentially important for selection and/or conservation and plotted against the position of the SNP across autosomes. Manhattan plots of the distribution of ROH in the four goat breeds (Egyptian Nubian, Damascus, Barki and Boer) showed the frequency (%) at which each SNP was observed in ROH across individuals (Fig. 6). Chromosome 18 had the highest peak in Egyptian Nubian, Barki and Boer breeds, but also chromosomes 6 showed high ROH occurrence in Damascus, Egyptian Nubian and Boer goats. Chromosomes 12, 13 and 19 presented high ROH peaks in one or more of the studied breeds. Applying the abovementioned threshold of 40%, 21 ROH hotspots were detected in total across all breeds (Table 2); the highest number of genomic regions identified was found in Egyptian Nubian breed with 11 ROH hotspots, followed by Boer, Barki and Damascus breed with 7, 2 and 1 ROH hotspots, respectively. The longest ROHs hotspots were found in Boer (1.5 Mb), followed by Egyptian Nubian (1.4 and 1.3 Mb), and Barki (1 Mb), while the shortest ROH was found in Damascus (~ 3 Kb). Genomic regions on Chromosome 18 (at 60 and 15 Mb) and Chromosome 6 (at 70 and 71 Mb) were found common in two or more breeds. The number of SNPs within these common regions ranged from 15 (Barki) to 22 (Boer) and from 6 (Damascus) to 37 (Egyptian Nubian) for the corresponding chromosomes, respectively.

**Table 2:** ROH peaks indicated by single nucleotide polymorphisms (SNP) occurring in an ROH in more than 40% of the individuals of the respective goat population

Breed	Start SNP	End SNP	Chr^1^	nSNP	From^2^	To^2^	Annotated genes^3^
Barki	snp30392-scaffold335-200469	snp30397-scaffold335-418126	12	6	50,047,521	50,253,456	*RNF17*
	snp54264-scaffold828-29085	snp15260-scaffold1625-7565	18	15	60,120,713	61,158,064	
Boer	snp27343-scaffold2904-5676	ilmnseq_rs119103308	18	15	15,731,287	16,105,667	*SPG7, CPNE7, SPATA2L, ZNF276, TCF25, MC1R*
	snp31127-scaffold3444-44647	GoatD01.018893	18	22	60,178,927	61,711,572	
	QTLSaanen19.36_O	QTLSaanen19.47_O	19	22	26,676,012	27,087,979	*PHF23, GABARAP, TMEM95, SPEM1, FGF11, TNFSF12, TNFSF13, CD68, SOX15*
	6_5343525_AF-PAKI	6_5343525_AF-PAKI	6	1	5,343,525	5,343,525	
	snp18690-scaffold189-404920	snp18687-scaffold189-286736	6	4	5,426,581	5,545,798	
	snp17777-scaffold1844-103982	Random2.2 K-637	6	7	6,514,342	6,755,014	
	ilmnseq_rs669256409	snp58095-scaffold94-5505002	6	16	70,744,246	70,834,128	*KIT*
Damascus	ilmnseq_rs662787102	ilmnseq_rs635694893	6	5	71,146,873	71,150,677	*KDR*
Egyptian Nubian	snp5758-scaffold1202-226615	snp15101-scaffold1613-34525	13	10	41,738,246	42,085,766	*CST3, CST7*
	snp56019-scaffold873-331410	snp59699-scaffold99-95183	18	22	15,776,603	16,198,866	*MC1R*
	snp30840-scaffold340-1887718	snp30815-scaffold340-824448	6	24	13,498,678	14,563,391	*ALPK1, TIFA*
	snp12525-scaffold148-867870	snp12531-scaffold148-1222465	6	6	31,990,015	32,349,183	
	snp12552-scaffold148-2118357	snp12570-scaffold148-2915803	6	18	33,249,084	34,054,242	
	snp12572-scaffold148-2999472	snp12580-scaffold148-3342396	6	10	34,138,098	34,484,359	
	snp26768-scaffold281-1436966	snp26799-scaffold281-2778330	6	31	38,091,565	39,437,063	
	snp7248-scaffold1268-14447	snp54008-scaffold821-914173	6	36	39,985,135	41,385,076	
	snp5662-scaffold1199-2118633	snp12889-scaffold1498-459920	6	26	44,424,892	45,863,398	*DHX15, PI4K2B*
	snp58085-scaffold94-5127553	snp58098-scaffold94-5630065	6	28	70,454,815	70,959,830	
	snp58100-scaffold94-5714186	snp58110-scaffold94-6171823	6	37	71,044,898	71,495,883	

^1^Chromosome, ^2^Location in base pairs, ^3^Based on the Ensembl database. *ROH *Runs of homozygosity

**Fig. 7:**
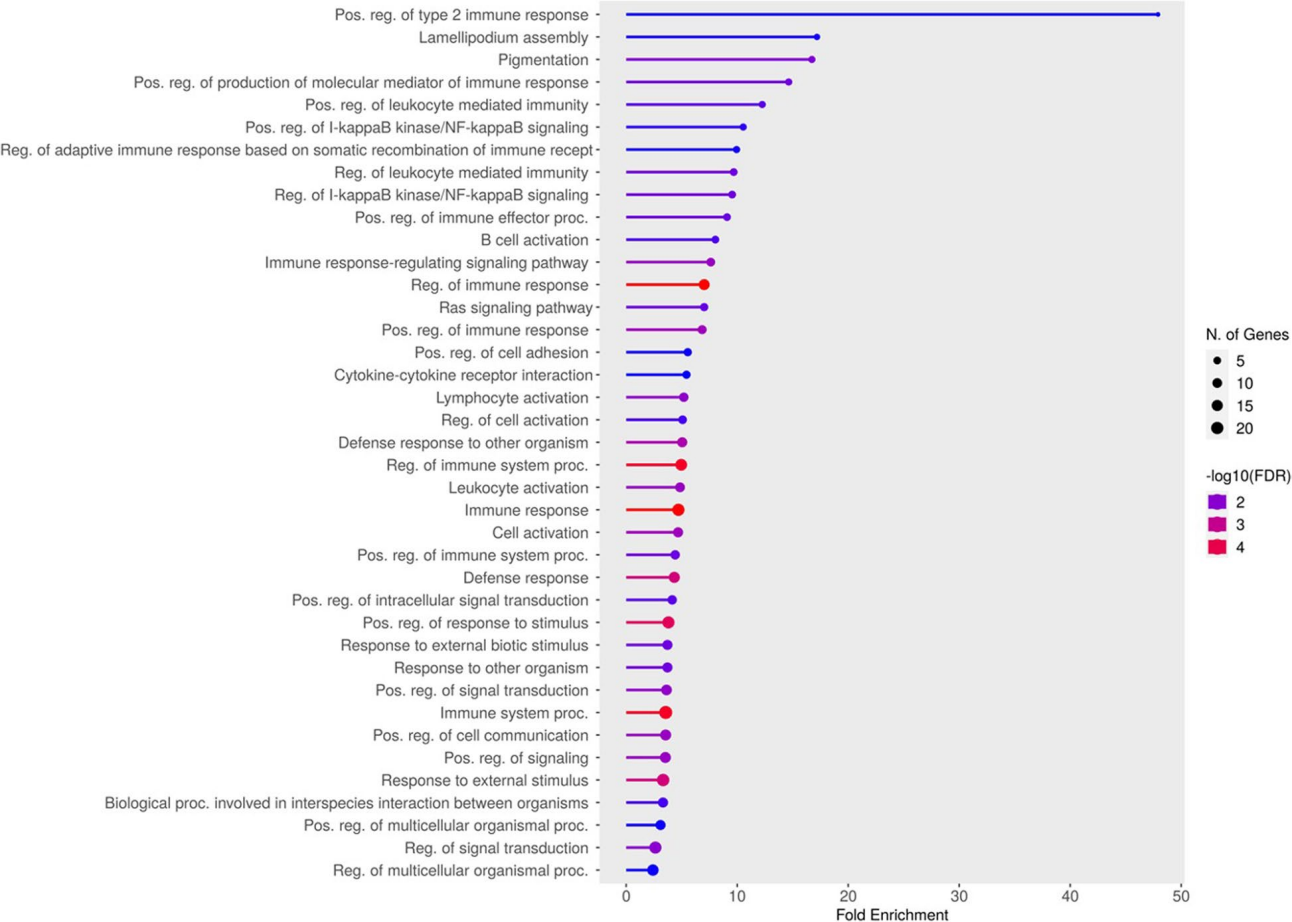
Gene ontology (GO) enrichment analysis of biological pathways for the list of candidate genes resulting from ROH and F_ST_ analyses in Egyptian goats. False discovery rate (FDR) < 0.05

The gene enrichment analysis identified 39 significantly enriched (FDR < 0.05) biological pathways using a list of the identified candidate genes in the current study (Supplementary excel file). The identified candidate genes were significantly enriched in biological pathways related to immunity and regulation of the immune response (Fig. 7); for instance, the positive regulation of type 2 immune response (FDR = 0.046) and pigmentation (FDR = 0.045).

### Genetic differentiation between breeds

The highest average F_ST_ between pairs of the breeds was 0.14 for Egyptian Nubian and Boer followed by 0.077 for Egyptian Nubian and Barki, 0.075 for Nubian and Damascus, 0.071 for Barki and Boer, 0.064 for Damascus and Boer, and the lowest was 0.015 for Damascus and Barki (Table 3). The SNP-specific F_ST_ values were further plotted against the autosomes of goat (Supplementary Figure S2). Analysis of breed specific differentiation between each pair of goat breeds included in the study resulted in several putative regions of selection and are presented in the Supplementary excel file. The top 10 SNPs that accounted for highest F_ST_ values between breeds in each of the pairwise comparisons are presented in Supplementary Tables S1, S2, S3, S4, S5 and S6. The SNP that accounted for the highest genetic differentiation (F_ST_ = 0.96) value was observed on chromosome 6 at 13.9 Mb (Nubian vs. Boer) while the SNP that accounted for the lowest genetic differentiation (F_ST_ = 0.35) was observed on chromosome 6 at 14.1 Mb (Damascus vs. Barki). Multiple overlapping SNPs residing in multiple ROH hotspots and F_ST_ genomic windows on two autosomes (chromosomes 6 and 18) was detected in the studied goat breeds.

**Table 3:** Pairwise F_ST_ estimates for all pairs of the studied goat breeds

	Egyptian Nubian	Damascus	Barki	Boer
**Egyptian Nubian**		0.075	0.077	0.14
**Damascus**			0.015	0.064
**Barki**				0.071

## Discussion

Considering the native habitat of Barki breed in the Egyptian desert, the breed was expected to cluster independently from the other breeds that predominate in the Nile Delta. Nonetheless, they have partially clustered together because of breeders’ efforts to improve growth and milk performance of Barki by crossing with Damascus [6]. The PCA results showed that Damascus and Boer goats are genetically connected, indicating gene flows between these breeds, in contrast to the Egyptian Nubian that was genetically distinct and has a separate genetic structure due to the application of specific breeding programs [52]. Boer goats were shown to be quite scattered in PCA plot despite implementing some sort of breeding programs. We speculate that this may be because those samples were taken from private farms, in which breeders apply uncontrolled crossing with Damascus, which has quite similar color and body characteristics as Boer. However, PCA clearly classified the four breeds based on SNPs information into distinct clusters. In agreement, Barki and Boer goats were differentiated in the PCA plot from the non-native breeds in independent clusters [5].

The average estimate of inbreeding measured by homozygosity (F_ROH_) showed higher values (0.12 and 0.11) for the breeds under intensive selection of important traits (Egyptian Nubian and Boer, respectively), compared to low values (0.05 and 0.03) for breeds that are not managed under specific breeding programs (Damascus and Barki, respectively) [53]. This shows that maintaining the local breeds with low population size is possible through adopting a breeding scheme that aim at less intensive selection for production traits [54], allowing a higher diversity in their genomes. The highest individual F_ROH_ estimate in the current study (F_ROH_ = 0.36) was higher than that reported in local Russian goat (F_ROH_ = 0.27). Likewise, the average F_ROH_ in the current study (F_ROH_ = 0.03 to 0.12) were higher than those reported in local Russian goats (F_ROH_ = 0.033 to 0.077) [55]. The average F_ROH_ in Nubian and Boer are lower than that estimated in Chinese goats (F_ROH_ = 0.13) [32] and consistent with those estimated at the worldwide scale in the global (F_ROH_ = 0.12) goat breeds [30]. Likewise, the average F_ROH_ in Barki (0.03) and Boer (0.11) in the current study were higher than those reported previously [5] for the same breeds (0.03 vs. 0.02 and 0.11 vs. 0.09), respectively. This may indicate that Damascus and Barki goats have higher genetic variability than Egyptian Nubian, Boer and the global goat breeds and therefore, have a greater chance to be conserved as a genetic resource [32].

The length of ROH regions is an important aspect of ROH as the longer ROHs refer to more recent inbreeding or artificial selection [7, 8], while shorter ROHs indicate ancient inbreeding [56]. The longest ROH category (> 16 Mb) was only present in Nubian goat, which indicate recent inbreeding and possible higher incidence of inbreeding depression in the future [56]. On the contrary, Damascus breed had the shortest mean length of ROH in their genomes. Interestingly, the shortest ROH segments were most common Boer goats in agreement with other studies of goats from Uganda [8], Russia [55], China [32] and Italy [57]. Furthermore, Kim et al. [5] reported larger ROHs length in Boer compared with Barki goats. There, the high frequency of shorter ROH segments in the breed genome was attributed to the presence of ancestral family relatedness [55].

The total number of ROH estimated in this study, which ranged from 30,544 (Egyptian Nubian) to 1846 (Barki) was lower than those detected in GX (*n* = 44,422) and GF (*n* = 16,598) Chinese goat breeds [32], while it was comparable to those identified in the Italian goat breeds (*n* = 28,383) [57]. Likewise, the average ROH number ranged from 145.44 (Nubian) to 42.02 (Damascus) and was lower than those detected in the corresponding Chinese breeds (*n* = 277.60 and 207.50), respectively [32]. In contrary, this number was higher than those reported in Italian goat breeds (*n* = 3.02–38.83) [58]. The analysis of patterns of homozygosity provides insight into demographic history [55]. The average accumulative ROH length estimated here in Egyptian Nubian (306 Mb) and Boer (261.7 Mb) were higher than those estimated in Turkish breeds (210.64 Mb), Central Asian breeds (260.64 Mb) [32], and Russian local breeds (79.42–183.94 Mb) [55].

The average ROH number estimated in the current study in Damascus (42.02 ROH) was lower than those in Turkish breeds (60 ROH) and Central Asian breeds (90 ROH) [30] [32], but higher than those detected in Russian local breeds (18–41 ROH) [55]. However, our study reported higher ROH numbers in Egyptian Nubian (145.44 ROH) and Boer (126.95 ROH) than those reported in the previous studies [59]. Moreover, Kim et al. [5] reported slightly shorter mean ROH length in Barki (1.7 vs. 1.48 Mb) and longer in Boer goats (2.06 vs. 10.61 Mb).

The studied breeds are dual-purpose breeds for dairy and meat production, so it was expected that the identified differentiated regions across breeds may contain more genes associated with such traits. However, most of the identified genes were related to immune response, adaptation and reproduction, which may imply that these animals tended to be selected for their higher adaptation performance as they live in extremely hard environmental condition characterized by shortage of feed supply and water resources, especially in desert [5, 8].

Within the identified top ROH hotspots, five genes were detected (CHR18, 15.73 Mb): *SPG7* (SPG7 Matrix AAA Peptidase Subunit, Paraplegin) and *CPNE7* (Copine 7) genes are playing a role in diverse cellular processes including membrane trafficking and intracellular motility [60, 61]; the *SPATA2L* (Spermatogenesis Associated 2 Like) gene is involved in sperm formation [62]; *TCF25* is a member of the basic helix-loop-helix (bHLH) family of transcription factors that are important in embryonic development and cell death control [63]; and the *MC1R* (Melanocortin1 Receptor) gene is known to be involved in pigmentation and hair color [64].

Another important ROH hotspot was detected on CHR19 at 26.67 Mb, which harbored candidate genes involved in **(1)** immunity: *PHF23* (PHD Finger Protein 23), *GABARAP* (GABA Type A Receptor-Associated Protein), *CD68* (CD68 Molecule) and *GPS2* (G Protein Pathway Suppressor2) [65–68], **(2)** sperm formation and reproduction: *TMEM95* (Transmembrane Protein 95), *SPEM1* (Spermatid Maturation 1) [69, 70], and **(3)** embryonic development, cell growth, morphogenesis, tissue repair and muscle growth: *FGF11* (Fibroblast Growth Factor 11) and *SOX15* (SRY-Box Transcription Factor 15) genes [71, 72].

The detection of genetic markers that influence reproduction traits (e.g., litter size) in goats has taken a lot of interest. Two candidate gene were detected on CHR6 at 70.74 and 71.14 Mb: **(1)** the *KIT* (KIT Proto-Oncogene, Receptor Tyrosine Kinase) gene, which plays an essential role in the regulation of cell survival and proliferation, hematopoiesis, stem cell maintenance, gametogenesis, mast cell development, migration and function, melanogenesis [73], and a possible pleotropic role in reproductive performance in livestock. The *KIT* and its ligand (i.e., *KITLG*), are two well-known pigmentation genes in livestock [52, 74], and selection signals have been detected in many different goat [75] and sheep populations [76]. Interestingly, the same gene was identified to differentiate between the Egyptian Nubian and Boer (F_ST_ = 0.89), which may explain their difference in the dominant hair colors. In addition to **(2)** the *KDR* (Kinase Insert Domain Receptor) gene is important in survival, migration and morphogenesis [77]. The *KDR* gene identified here in Egyptian Nubian and Damascus breeds was previously reported in wild and domestic sheep [28] and was associated with proliferation, survival, migration, and tubular morphogenesis in mammals.

Importantly, the same candidate genes and genomic regions on CHR6 (70.74 Mb, *KIT*) and CHR18 (15.73 Mb, *SPG*) were recently identified by whole genome sequencing as top ROH hotspots in Jianchang Black goats [52]. Furthermore, the *RNF17* (Ring Finger Protein 17) gene that encodes a testis-specific protein containing a RING finger domain and may be involved in spermiogenesis [78] was detected on CHR12 (50.04 Mb).

Two candidate genes that are playing a role in immune regulation were identified on CHR13 (41.73 Mb): *CST3* (Cystatin C) and *CST7* (Cystatin F) [79, 80]. Additionally, two candidate genes were identified on CHR6 (13.49 Mb): *ALPK1* (Alpha Kinase 1) and *TIFA* (TRAF Interacting Protein with Forkhead Associated Domain), which are involved in the innate immune response, adaptive immunity and DNA damage response [81]. The 44.42 Mb on CHR6 harbors the *DHX15* (DEAH-Box Helicase 15) and *PI4K2B* (Phosphatidylinositol 4-Kinase Type 2 Beta) genes, which play a key role in antiviral innate immunity [82] and early T cell activation [83], respectively.

A considerable overlap of selection signatures was observed between results of ROH and F_ST_ approaches implemented in the current study. The common variants from both approaches were observed on CHR6 (at 13.49, 34.13, and 70.74 Mb) and CHR18 (at 15.73 and 60.12 Mb). The region on CHR6 at 13.49 Mb (F_ST_ = 0.74) harbored two immunity-relate genes: *TIFA* (TRAF Interacting Protein with Forkhead Associated Domain) and ALPK1 (Alpha Kinase 1). The region on CHR6 at 44.42 Mb (F_ST_ = 0.69) harbored two potential genes, *PI4K2B* (Phosphatidylinositol 4-Kinase Type 2 Beta) and *CCK1* (cholecystokinin1 receptor) that are involved in the regulation of vesicular trafficking [84] and food intake [85], respectively. The same region was reported by Kim et al. [5] in multiple goat breeds including Barki and Boer.

The F_ST_ value estimated in the pairwise comparison of Egyptian Nubian vs. Barki (F_ST_ = 0.077) was lower than reported by Agha et al. [86] using microsatellite markers (F_ST_ = 0.17). The high overlap observed between F_ST_ and ROH results suggests that the selection on genes related to immunity and adaptation is not breed-specific [8]. Multiple overlapping SNPs residing in multiple ROH hotspots and F_ST_ genomic windows on two autosomes (CHR6 and 18) between the studied goat populations were detected. Interestingly, one ROH hotspot on CHR12 (at 50.04 Mb) was common in the global goat breeds [30], which harbored genes related to vision (*GJA3*) and hearing (*GJB2* and *GJB6*) and are essential for adaptive evolution in goats [32].

The pairwise differentiation identified genetic variants harboring candidate genes that may control the breed-specific traits. For instance, three potential candidate genes appeared to differentiate between Damascus and Boer located at 31.98 Mb on CHR29 (F_ST_ = 0.68): **(1)***ETS1* (ETS Proto-Oncogene1), which is involved in stem cell development and death and the differentiation, survival and proliferation of lymphoid cells [87], **(2)***IGSF9B* (Immunoglobulin Superfamily Member 9B), which is involved in immune response [88], and **(3)***SPATA19* (Spermatogenesis Associated 19), which plays an important role in sperm motility and correct sperm midpiece assembly [89].

The region at 36.49 Mb on CHR6 harbored three candidate genes to differentiate between Egyptian Nubian and Barki (F_ST_ = 0.68) and Nubian and Boer (F_ST_ = 0.95): **(1)***HERC5* (HECT And RLD Domain Containing E3 Ubiquitin Protein Ligase 5), which is important in the antiviral immune response [90], **(2)***PPM1K* (Protein Phosphatase, Mg2+/Mn2 + Dependent 1 K), which is essential for cellular survival and development [91], and **(3)***SPP1* (Secreted Phosphoprotein 1), which acts as a cytokine involved in enhancing production of interferon-gamma and interleukin-12 and reducing production of interleukin-10 [92].

Results of GO and KEGG for the list of candidate genes identified in the current study support the assumption that pathways associated with adaptation mechanisms to harsh environmental conditions, such as regulation of immune response may be under positive selection in Egyptian goat breeds. These findings agree with previous reports in Egyptian [5] and Ugandan [8] goat breeds and indicates that adaptation to local environmental conditions requires a complex network of multiple genes [93], which is an important feature of indigenous breeds to increase their adaptation capacity.

Summarizing, a detailed picture of the genetic structure and the ROH landscape of Egyptian Nubian, Barki, Damascus, and Boer goat breeds was given using SNP markers. The investigated animals came from different breeding systems and geographical backgrounds, in which different selection processes are applied. Additionally, the ROH hotspots revealed that many candidate genes are putatively under selection for adaptation, immunity, growth and reproduction in the investigated breeds, which is compatible with our previous rationale hypothesis.

## Conclusion

Multiple signatures of selection were detected in four goat breeds in Egypt using runs of homozygosity (ROH) and fixation index (F_ST_) approaches. The identified chromosomal regions harbor candidate genes that encode proteins involved in the innate and adaptive immunity, vesicular trafficking, feed intake, sperm motility, cell development and embryonic growth and development, and adaptation. This implies that the genomes of Egyptian local goat breeds are shaped by adaptation to hard arid environmental conditions. Our results revealed recent genomic inbreeding in the local Egyptian Nubian goat, probably due to closed breeding in small populations, with possible inbreeding depression in the future. To protect this precious goat germplasm resource in Egypt, more scientific conservation strategies and efficient management systems should be established. Our findings provided a better understanding of the genomic landscape and evolutionary history of local Egyptian goat breeds and will benefit future breeding programs in local goat.

### Supplementary Information

**Additional file 1: Figure S1. **Pictures of the investigated goats in the current study. **Figure S2.** Manhattan plots for SNP-specific pairwise fixation index (*F*_ST_). Genetic differentiation between individuals of four goat breeds in Egypt(Nubian, Damascus, Barki and Boer). *F*_ST_ estimates are represented on the x-axis and genomic positions on chromosomes on y-axis. Each dot represents a SNP.

**Additional file 2.**

**Additional file 3: Table S1. **The top 10 single nucleotide polymorphisms (SNP) and annotated genes differentiating between Egyptian Nubian and Damascus goat breeds based on F_ST_ estimates.^1^Chromosome, ^2^Location in base pairs, ^3^Minor allele frequency, ^4^Based on the Ensembl database. **Table S2. **The top 10 single nucleotide polymorphisms (SNP) and annotated genes differentiating between Egyptian Nubian and Boer goat breeds based on F_ST_ estimates.^1^Chromosome,^2^Location in base pairs, ^3^Minor allele frequency, ^4^Based on the Ensembl database.**Table S3. **The top 10 single nucleotide polymorphisms (SNP) and annotated genes differentiating between Egyptian Nubian and Barki goat breeds based on F_ST_ estimates.^1^Chromosome, ^2^Location in base pairs, ^3^Minor allele frequency, ^4^Based on the Ensembl database. **Table S4. **The top 10 single nucleotide polymorphisms (SNP) and annotated genes differentiating between Damascus and Boer goat breeds based on F_ST_ estimates.^1^Chromosome, ^2^Location in base pairs, ^3^Minor allele frequency, ^4^Based on the Ensembl database. **Table S5. **The top 10 single nucleotide polymorphisms (SNP) and annotated genes differentiating between Damascus and Barki goat breeds based on F_ST_ estimates.^1^Chromosome,^2^Location in base pairs, ^3^Minor allele frequency, ^4^Based on the Ensembl database.**Table S6. **The top 10 single nucleotide polymorphisms (SNP) and annotated genes differentiating between Barki and Boer goat breeds based on F_ST_ estimates. ^1^Chromosome,^2^Location in base pairs, ^3^Minor allele frequency, ^4^Based on the Ensembl database.

## Data Availability

The datasets generated and/or analysed during the current study are available in the OSF repository using the following link: https://osf.io/7q6j8/ and can be downloaded upon request from the corresponding author (ahmedsallam2@gmail.com).
