# Supplementary material for: Genome-wide landscape of runs of homozygosity and differentiation across Egyptian goat breeds
Source: BMC Genomics. 2023 Sep 26;24:573. doi: 10.1186/s12864-023-09679-6 (PMC10521497; doi:10.1186/s12864-023-09679-6)
Supplement: Supplementary file 1 — Additional file 1: Figure S1. Pictures of the investigated goats in the current study. Figure S2. Manhattan plots for SNP-specific pairwise fixation index (FST). Genetic differentiation between individuals of four goat breeds in Egypt(Nubian, Damascus, Barki and Boer). FST estimates are represented on the x-axis and genomic positions on chromosomes on y-axis. Each dot represents a SNP. [file 12864_2023_9679_MOESM1_ESM.docx]

**Supplementary Figures**

**
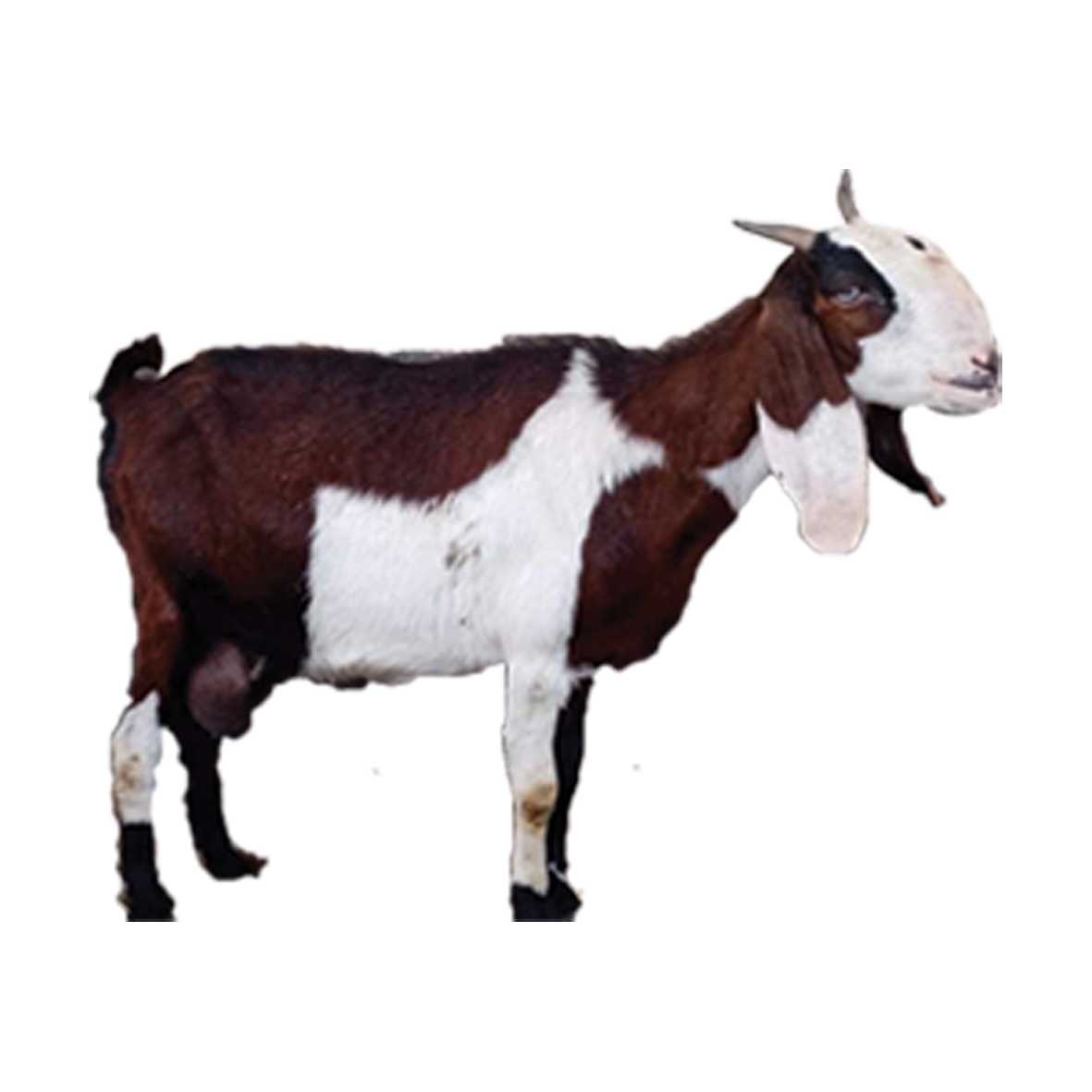
**

**
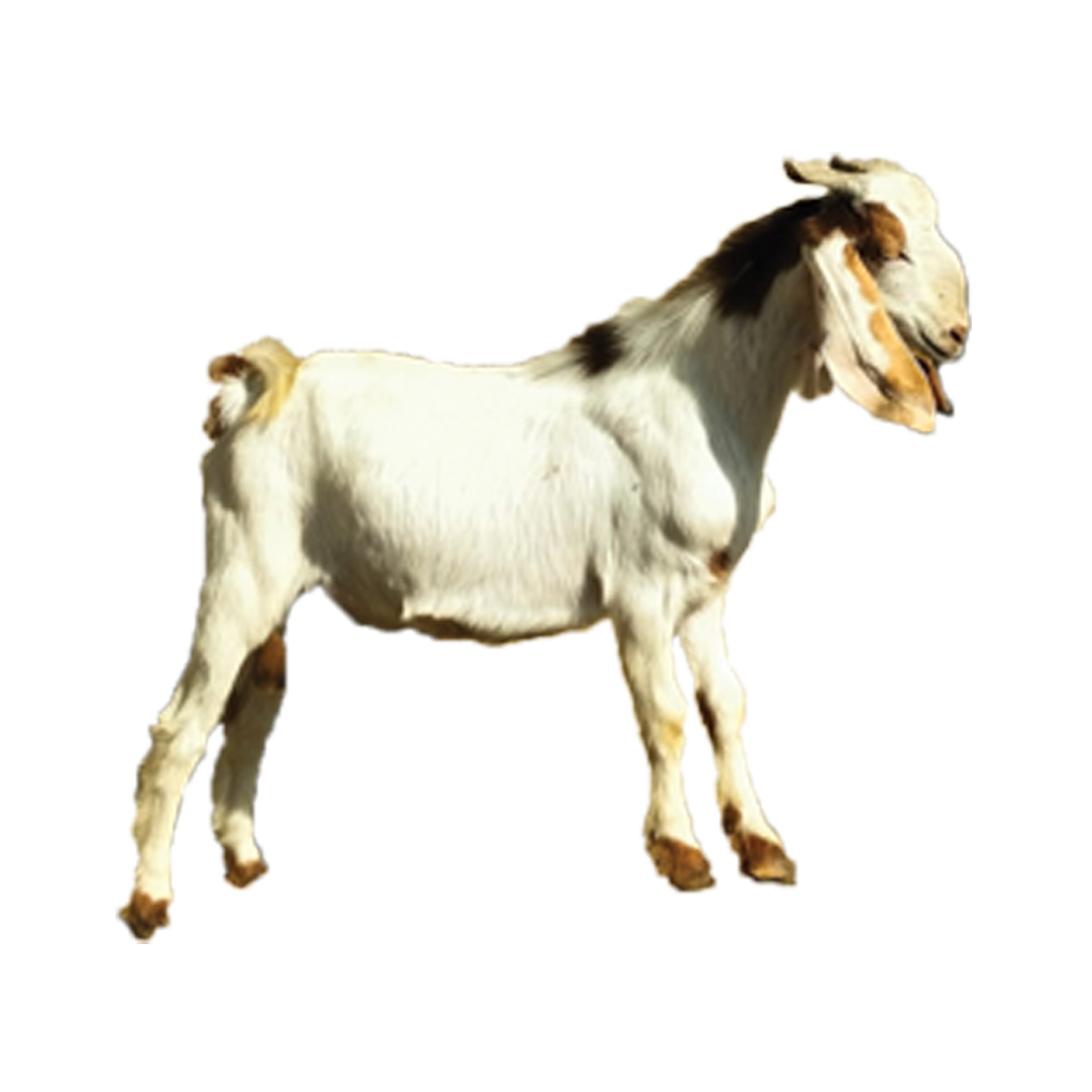
**

**Egyptian Nubian goat**

**
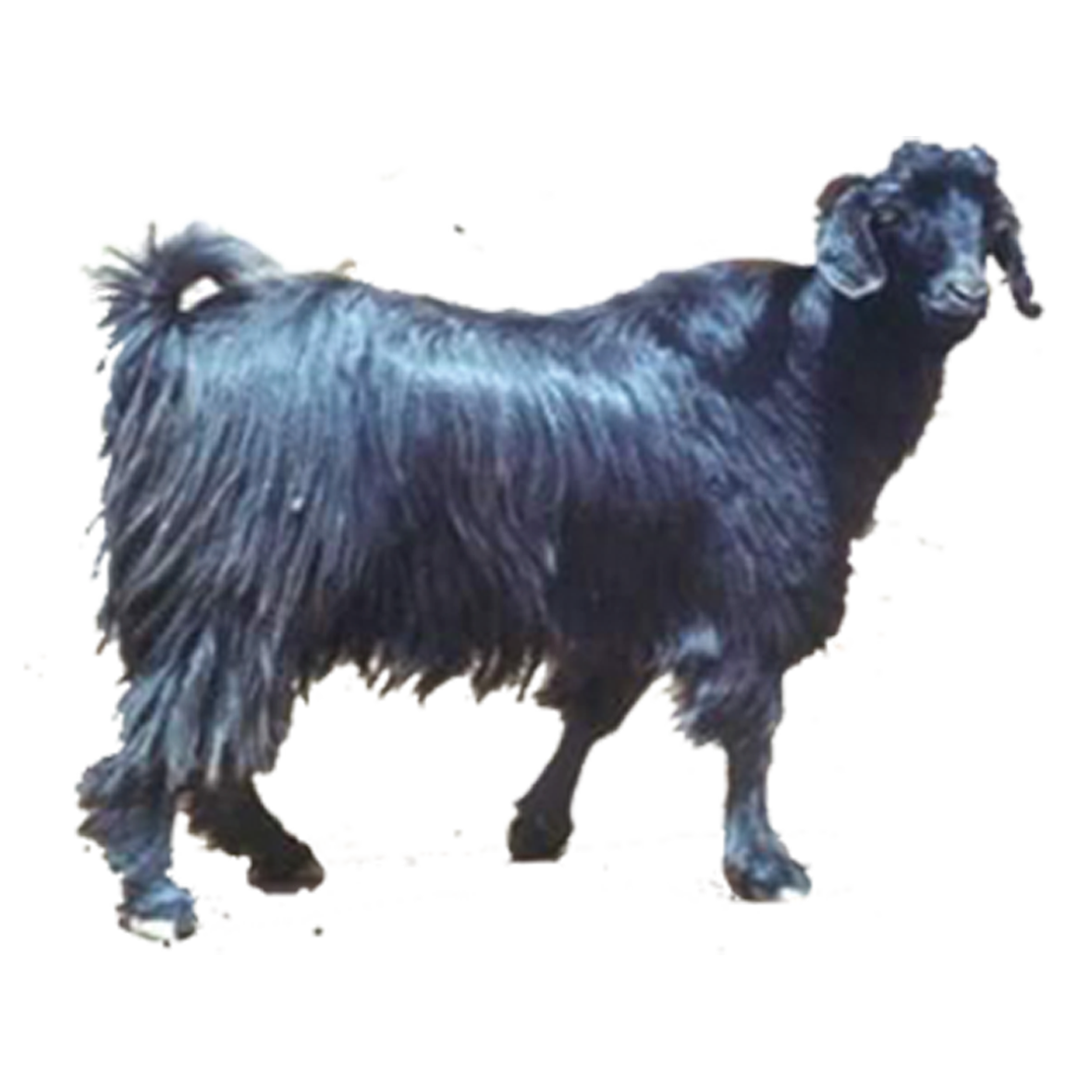
**

**Barki goat**

**
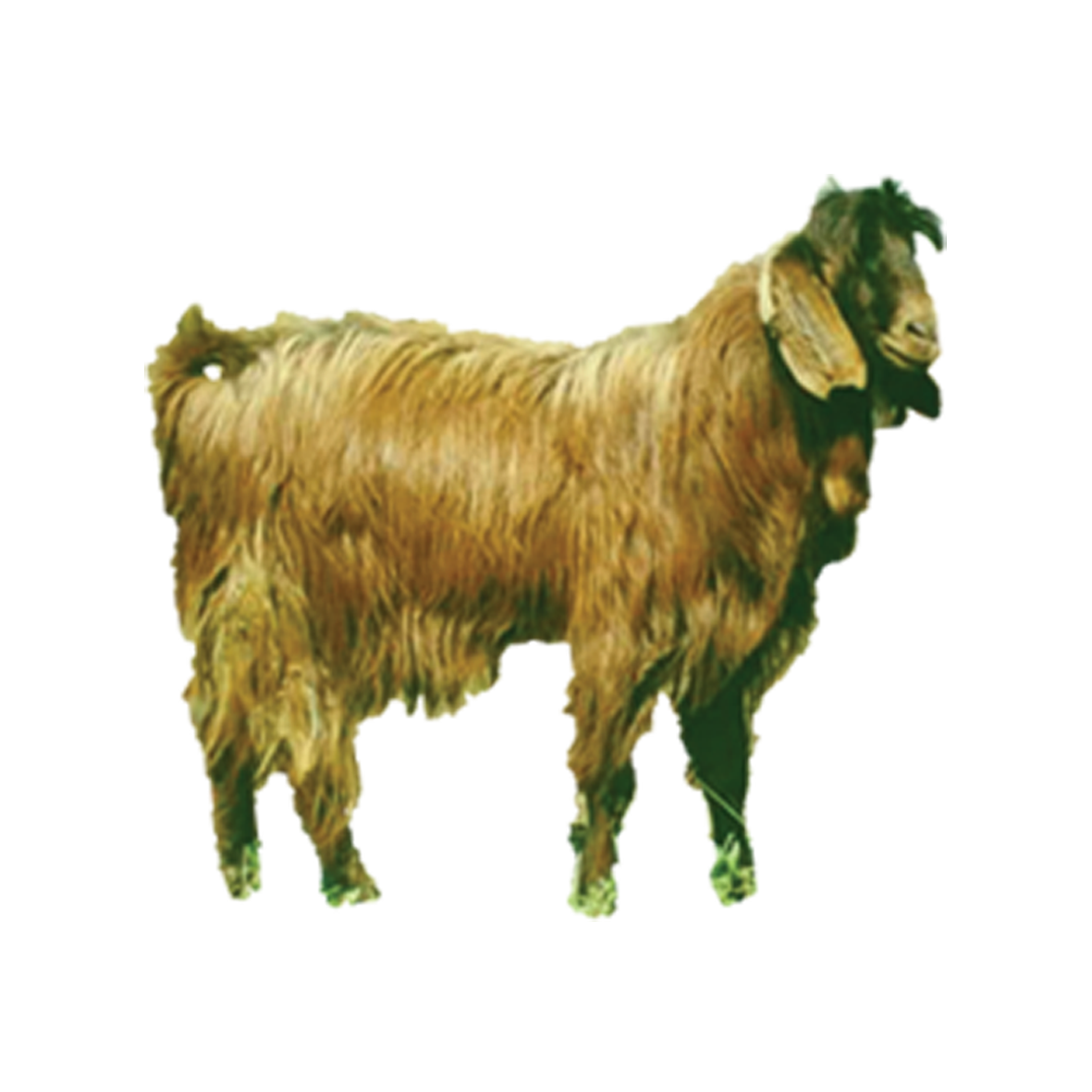
**

**Damascus goat**

**
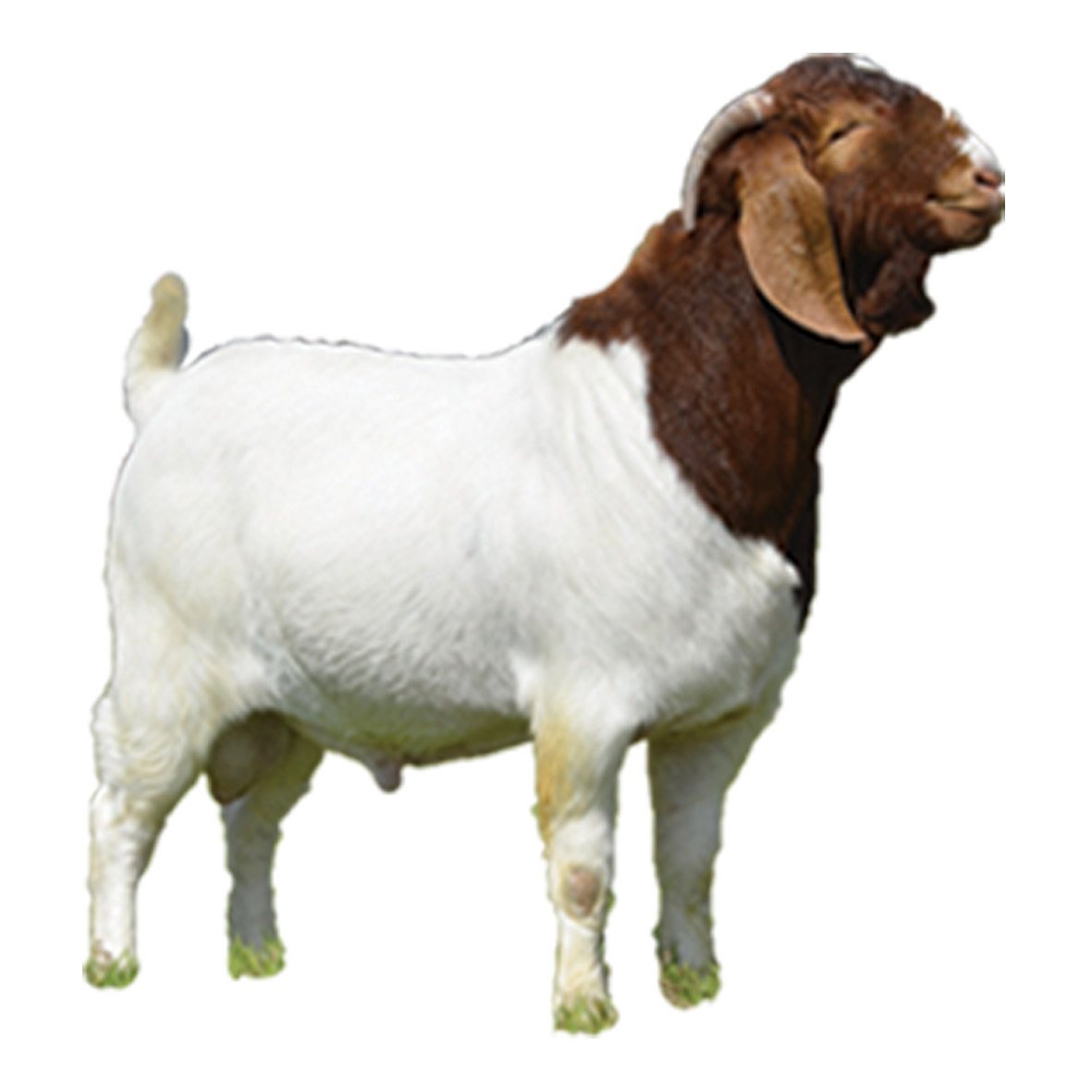
**

**Boer goat**

**Figure S1.** Pictures of the investigated goats in the current study.

**
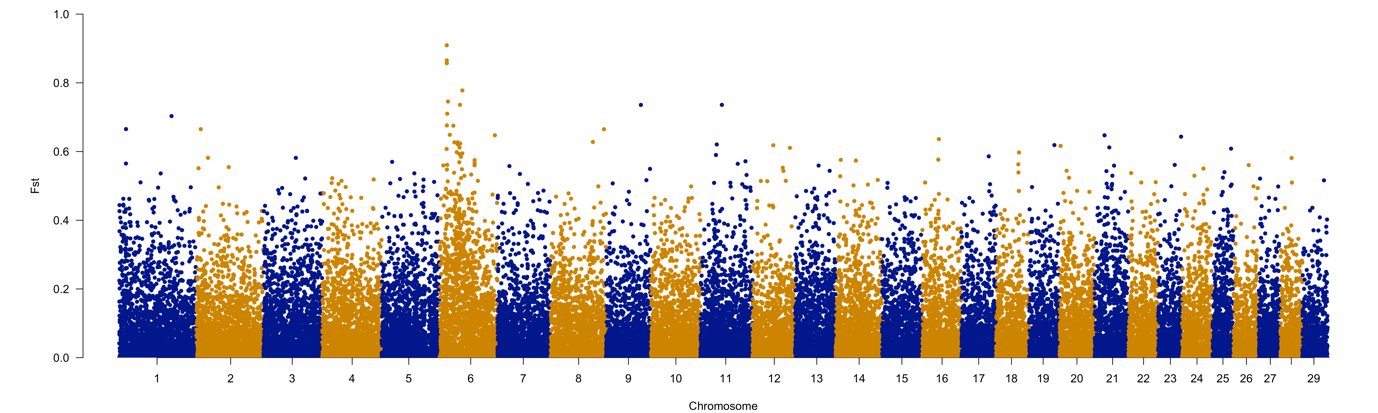
Nubian x Damascus**

**
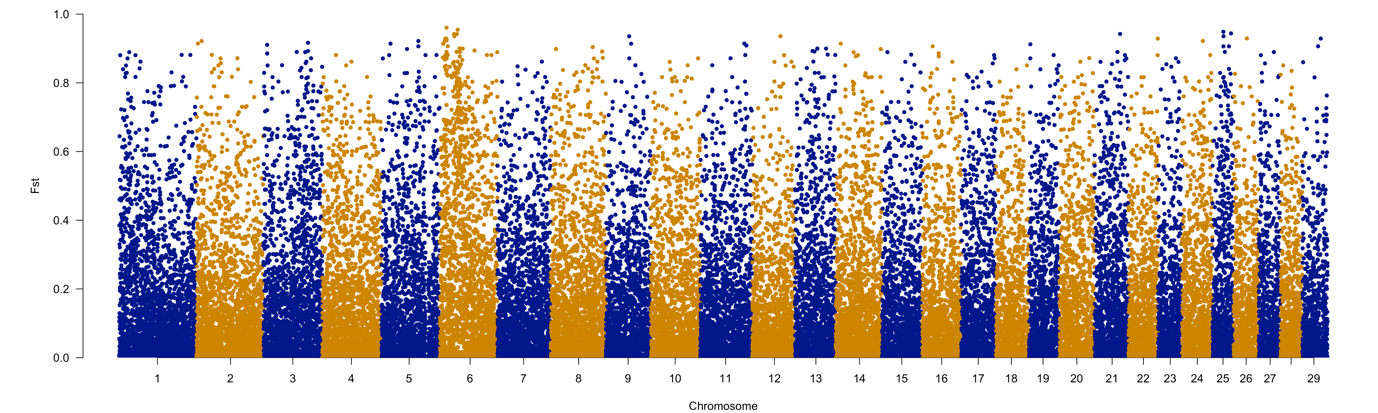
Nubian x Boer**

**
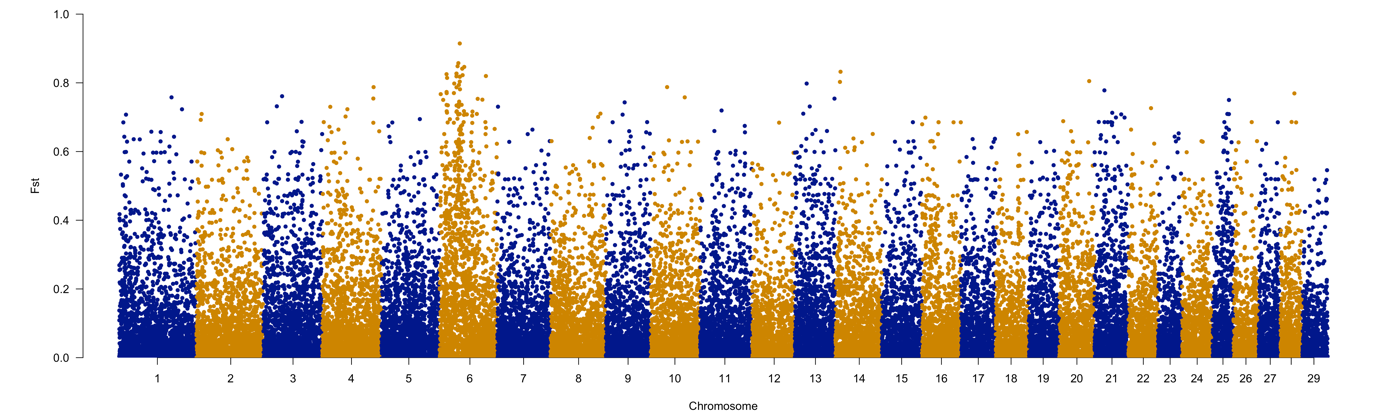
Nubian x Barki**

**
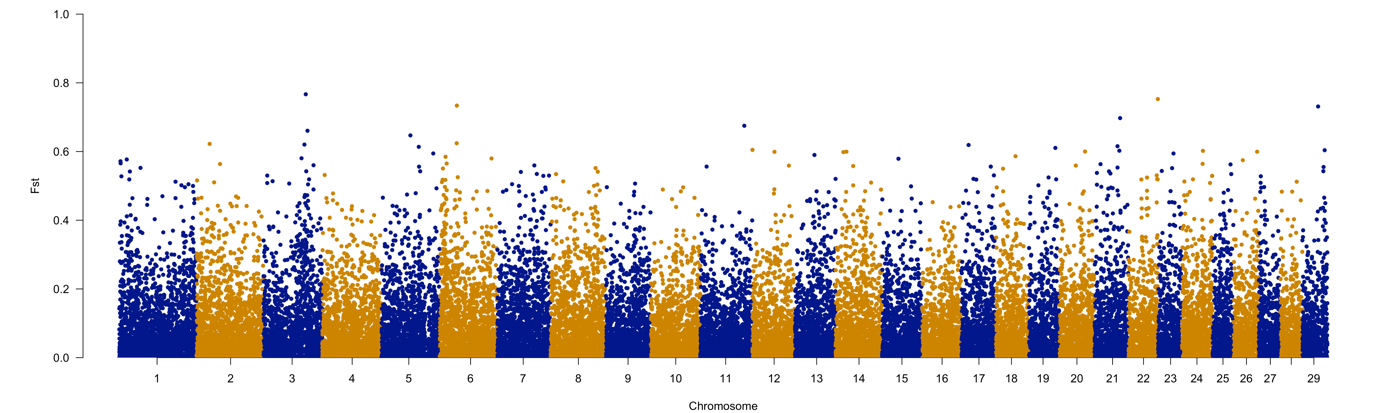
Damascus x Boer**

**Damascus x Barki**

**
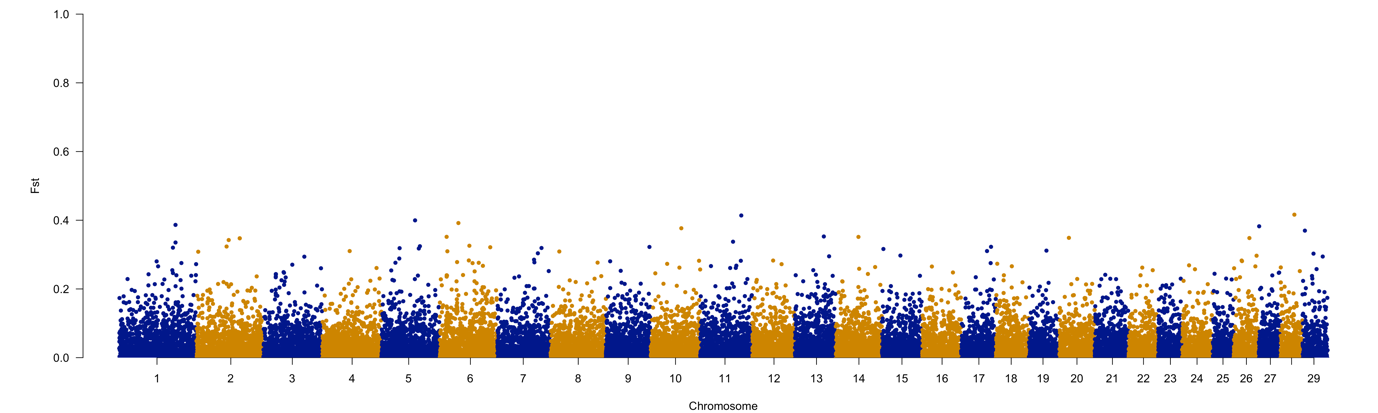
**

**Barki x Boer**

**
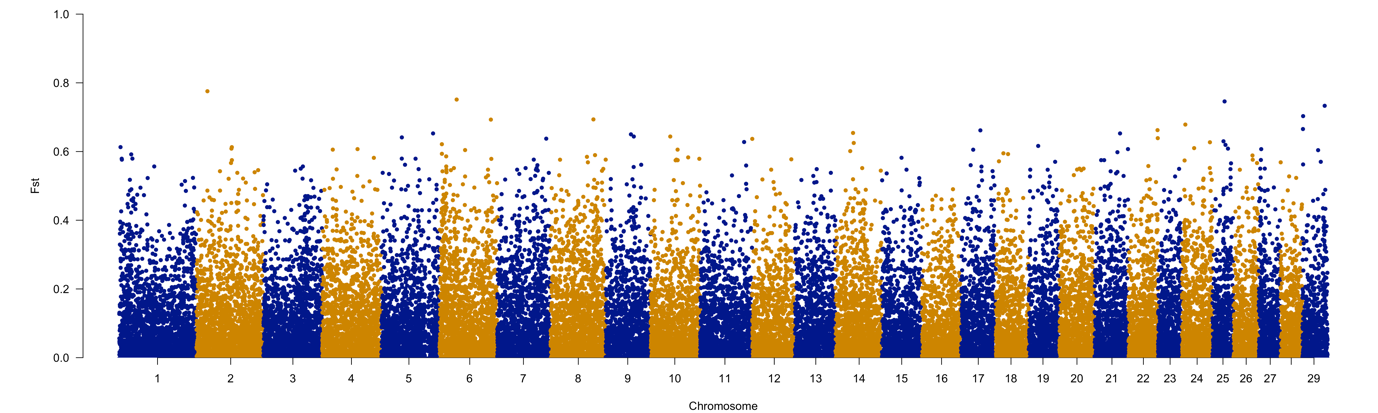
**

**Figure S2.** Manhattan plots for SNP-specific pairwise fixation index (*F_ST_*). Genetic differentiation between individuals of four goat breeds in Egypt (Nubian, Damascus, Barki and Boer). *F_ST_* estimates are represented on the x-axis and genomic positions on chromosomes on y-axis. Each dot represents a SNP.
