# Supplementary material for: Genome-wide landscape of runs of homozygosity and differentiation across Egyptian goat breeds
Source: BMC Genomics. 2023 Sep 26;24:573. doi: 10.1186/s12864-023-09679-6 (PMC10521497; doi:10.1186/s12864-023-09679-6)
Supplement: Supplementary file 3 — Additional file 3: Table S1. The top 10 single nucleotide polymorphisms (SNP) and annotated genes differentiating between Egyptian Nubian and Damascus goat breeds based on FST estimates.1Chromosome, 2Location in base pairs, 3Minor allele frequency, 4Based on the Ensembl database. Table S2. The top 10 single nucleotide polymorphisms (SNP) and annotated genes differentiating between Egyptian Nubian and Boer goat breeds based on FST estimates.1Chromosome,2Location in base pairs, 3Minor allele frequency, 4Based on the Ensembl database.Table S3. The top 10 single nucleotide polymorphisms (SNP) and annotated genes differentiating between Egyptian Nubian and Barki goat breeds based on FST estimates.1Chromosome, 2Location in base pairs, 3Minor allele frequency, 4Based on the Ensembl database. Table S4. The top 10 single nucleotide polymorphisms (SNP) and annotated genes differentiating between Damascus and Boer goat breeds based on FST estimates.1Chromosome, 2Location in base pairs, 3Minor allele frequency, 4Based on the Ensembl database. Table S5. The top 10 single nucleotide polymorphisms (SNP) and annotated genes differentiating between Damascus and Barki goat breeds based on FST estimates.1Chromosome,2Location in base pairs, 3Minor allele frequency, 4Based on the Ensembl database.Table S6. The top 10 single nucleotide polymorphisms (SNP) and annotated genes differentiating between Barki and Boer goat breeds based on FST estimates. 1Chromosome,2Location in base pairs, 3Minor allele frequency, 4Based on the Ensembl database. [file 12864_2023_9679_MOESM3_ESM.docx]

**Table S1.** The top 10 single nucleotide polymorphisms (SNP) and annotated genes differentiating between Egyptian Nubian and Damascus goat breeds based on F_ST_ estimates.

| **SNP_ID** | **Chr**^1^ | **Location**^2^ | ***F_st_*** | **Annotated genes**^4^ |
| --- | --- | --- | --- | --- |
| snp30825-scaffold340-1270511 | 6 | 14116546 | 0.90 | *TIFA, ALPK1* |
| snp30823-scaffold340-1209238 | 6 | 14178198 | 0.86 |  |
| snp30821-scaffold340-1076911 | 6 | 14310591 | 0.85 |  |
| snp12891-scaffold1498-560289 | 6 | 45963557 | 0.85 | *PI4K2B, RBPJ, CCK1* |
| snp52924-scaffold795-486047 | 6 | 16637583 | 0.77 | *ELOVL6, RRH, CFI, PLA2G12A, MCUB, SEC24B* |
| snp7624-scaffold1273-248604 | 6 | 40982644 | 0.74 | *KCNIP4* |
| snp24326-scaffold247-6085225 | 11 | 43596318 | 0.73 | *REL, EVA1A, EDAR* |
| snp8445-scaffold1304-1378829 | 9 | 71031168 | 0.73 | *RAB32, ADGB* |
| snp30802-scaffold340-250189 | 6 | 15137588 | 0.71 | *ELOVL6, PITX2* |
| snp46689-scaffold65-2302839 | 1 | 106428851 | 0.70 | *ARL14, TRIM59, IL12A, SCHIP1* |

^1^Chromosome, ^2^Location in base pairs, ^3^Minor allele frequency, ^4^Based on the Ensembl database

**Table S2.** The top 10 single nucleotide polymorphisms (SNP) and annotated genes differentiating between Egyptian Nubian and Boer goat breeds based on F_ST_ estimates.

| **SNP_ID** | **Chr**^1^ | **Location**^2^ | ***F_st_*** | **Annotated genes**^4^ |
| --- | --- | --- | --- | --- |
| snp30829-scaffold340-1484914 | 6 | 13901585 | 0.96 | *TIFA, ALPK1* |
| snp16820-scaffold1760-823922 | 6 | 36498331 | 0.95 | *HERC5, PPM1K, SPP1* |
| snp23682-scaffold239-1377449 | 25 | 22138261 | 0.94 | *PRKCB, CACNG3, SLC5A11, AQP8* |
| snp29752-scaffold3220-348924 | 25 | 37715697 | 0.94 | *PI4K2B, RBPJ, CCK1* |
| snp14076-scaffold156-39128 | 21 | 52040333 | 0.94 | *AZGP1, GJC3, OR2AE1, CYP3A43, TMEM225B* |
| snp58984-scaffold968-854575 | 6 | 34831119 | 0.94 | *-* |
| snp58984-scaffold968-854575 | 6 | 29203826 | 0.94 | *-* |
| snp23676-scaffold239-1132860 | 25 | 22383234 | 0.93 | *PRKCB, CACNG3, SLC5A11, AQP8* |
| snp58996-scaffold968-1405713 | 6 | 29759230 | 0.93 | *-* |
| snp50252-scaffold717-7820456 | 12 | 57340321 | 0.93 | *-* |

^1^Chromosome, ^2^Location in base pairs, ^3^Minor allele frequency, ^4^Based on the Ensembl database

**Table S3.** The top 10 single nucleotide polymorphisms (SNP) and annotated genes differentiating between Egyptian Nubian and Barki goat breeds based on F_ST_ estimates.

| **SNP_ID** | **Chr**^1^ | **Location**^2^ | ***F_st_*** | **Annotated genes**^4^ |
| --- | --- | --- | --- | --- |
| snp7624-scaffold1273-248604 | 6 | 40982644 | 0.91 | *KCNIP4* |
| snp26768-scaffold281-1436966 | 6 | 38091565 | 0.85 | *-* |
| snp16820-scaffold1760-823922 | 6 | 36498331 | 0.84 | *HERC5, PPM1K, SPP1* |
| snp17109-scaffold1789-197608 | 6 | 50092811 | 0.84 | *-* |
| snp12891-scaffold1498-560289 | 6 | 45963557 | 0.84 | *SLC34A2* |
| snp8380-scaffold1300-860783 | 14 | 10302583 | 0.83 | *TRIQK* |
| 6_34193381_AF-PAKI | 6 | 34193381 | 0.82 | *-* |
| snp12577-scaffold148-3245543 | 6 | 34386561 | 0.82 | *-* |
| snp30827-scaffold340-1374445 | 6 | 14012807 | 0.82 | *TIFA, ALPK1* |
| snp52124-scaffold771-805954 | 6 | 46773590 | 0.82 | *-* |

^1^Chromosome, ^2^Location in base pairs, ^3^Minor allele frequency, ^4^Based on the Ensembl database

**Table S4.** The top 10 single nucleotide polymorphisms (SNP) and annotated genes differentiating between Damascus and Boer goat breeds based on F_ST_ estimates.

| **SNP_ID** | **Chr**^1^ | **Location**^2^ | ***F_st_*** | **Annotated genes**^4^ |
| --- | --- | --- | --- | --- |
| snp25327-scaffold261-524798 | 3 | 86188582 | 0.76 | *VAV3, SLC25A24, CLCC1* |
| Random2.2K-2022 | 22 | 59574735 | 0.75 | *-* |
| snp12587-scaffold148-3686817 | 6 | 34831119 | 0.73 | *-* |
| snp21645-scaffold2119-513161 | 29 | 31985352 | 0.73 | *ETS1, IGSF9B, SPATA19* |
| snp14076-scaffold156-39128 | 21 | 52040333 | 0.69 | *-* |
| snp29754-scaffold3229-24398 | 11 | 89664079 | 0.67 | *RSAD2, RNF144A, SOX11* |
| snp47949-scaffold675-3536858 | 3 | 89708973 | 0.66 | *KCND3, MOV10* |
| snp42470-scaffold562-593775 | 5 | 59091840 | 0.64 | *TESPA1* |
| snp12581-scaffold148-3383733 | 6 | 34526728 | 0.62 | *-* |
| snp7415-scaffold127-1573416 | 2 | 26800016 | 0.62 | *-* |

^1^Chromosome, ^2^Location in base pairs, ^3^Minor allele frequency, ^4^Based on the Ensembl database

**Table S5.** The top 10 single nucleotide polymorphisms (SNP) and annotated genes differentiating between Damascus and Barki goat breeds based on F_ST_ estimates.

| **SNP_ID** | **Chr**^1^ | **Location**^2^ | ***F_st_*** | **Annotated genes**^4^ |
| --- | --- | --- | --- | --- |
| snp54846-scaffold838-3479273 | 28 | 28210425 | 0.41 | *CABCOCO1, RTKN2* |
| snp33715-scaffold396-836773 | 11 | 83613145 | 0.41 | *LRATD1* |
| snp28841-scaffold310-5804323 | 5 | 68676723 | 0.39 | *TMEM263* |
| snp26768-scaffold281-1436966 | 6 | 38091565 | 0.39 | *-* |
| snp398-scaffold1009-1333513 | 1 | 114514997 | 0.38 | *-* |
| snp2229-scaffold1068-844565 | 27 | 1051338 | 0.38 | *-* |
| snp33159-scaffold388-1292888 | 10 | 62245890 | 0.37 | *-* |
| snp13770-scaffold1537-171977 | 29 | 5086848 | 0.36 | *-* |
| snp48699-scaffold691-2724642 | 13 | 59166263 | 0.35 | *TFAP2C, SLC52A3* |
| snp30825-scaffold340-1270511 | 6 | 14116546 | 0.35 | *TIFA, ALPK1* |

^1^Chromosome, ^2^Location in base pairs, ^3^Minor allele frequency, ^4^Based on the Ensembl database

**Table S6.** The top 10 single nucleotide polymorphisms (SNP) and annotated genes differentiating between Barki and Boer goat breeds based on F_ST_ estimates.

| **SNP_ID** | **Chr**^1^ | **Location**^2^ | ***F_st_*** | **Annotated genes**^4^ |
| --- | --- | --- | --- | --- |
| snp7520-scaffold127-6031408 | 2 | 22317281 | 0.77 | *DOCK10* |
| snp12587-scaffold148-3686817 | 6 | 34831119 | 0.75 | *-* |
| snp50760-scaffold734-1084855 | 25 | 24595028 | 0.74 | *IL4R, IL21R* |
| snp23333-scaffold2330-462960 | 29 | 45374826 | 0.73 | *PELI3, BBS1, CCDC87, RHOD* |
| snp14782-scaffold1596-378830 | 29 | 1444738 | 0.70 | *-* |
| snp19314-scaffold1950-30244 | 8 | 87524484 | 0.69 | *-* |
| snp48631-scaffold690-3758133 | 6 | 104531980 | 0.69 | *LYAR, OTOP1* |
| snp834-scaffold1022-261355 | 24 | 6836509 | 0.67 | *-* |
| snp14775-scaffold1596-57767 | 29 | 1130343 | 0.66 | *-* |
| snp56004-scaffold870-706630 | 22 | 59106364 | 0.66 | *-* |

^1^Chromosome, ^2^Location in base pairs, ^3^Minor allele frequency, ^4^Based on the Ensembl database
